# Structure, evolution, phylogeny, and analysis of domain-deficient genes in the *IQD* gene family of *Brassica juncea*

**DOI:** 10.1038/s41598-026-42340-2

**Published:** 2026-03-02

**Authors:** Yan Hu, Xinyue Song, Xiaqin Chen, Shanshan Liu, Yuyu Dang, Yalin Qin, Hui Ling, Weishe Hu

**Affiliations:** 1 College of Agriculture and biotechnology, Hunan University of Humanities, Science and Technology, Loudi, 417000 Hunan China; 2 Key Laboratory of Development, Utilization, Quality and Safety Control of Characteristic Agricultural Resources in Central Hunan Province, Loudi, 417000 Hunan China; 3 College of Agriculture and Biology, Liaocheng University, Liaocheng, 252059 Shandong China

**Keywords:** Brassica juncea, *IQD* gene family, Evolutionary analysis, Expression analysis, Pseudogenes, Zn stress, Genetics, Molecular biology, Plant sciences

## Abstract

The plant *IQD* gene family plays crucial roles in abiotic stress response and plant growth regulation. However, the biological functions of *IQD* genes in *Brassica juncea* remain largely unknown. Here, we conducted biological analyses to identify and characterize the *IQD* gene family in *B. juncea*, providing new insights for future research on the *IQD* gene family. We identified 107 *IQD* genes in *B. juncea*, which are distributed across 18 chromosomes. Collinearity analysis reveals that segmental duplication was the primary mode of replication for *IQD* genes during evolution. The Ka/Ks ratio indicates that the *BjIQD* genes underwent strong purifying selection during evolution. GO functional annotation analysis shows that protein binding, tubulin binding, and microtubule binding are the most enriched GO terms. qRT-PCR analysis reveals that the *BjIQD* genes are regulated in response to Zn stress. Furthermore, some genes lacking the key IQ and DUF4005 domains were analyzed, and we hypothesize that these domain-deficient genes may still perform certain biological functions. This study analyzed the *B. juncea IQD* gene family from multiple perspectives and explored members lacking key domains. These findings provide insights into the evolution of the *BjIQD* genes and the analysis of different gene families.

**Supplementary Information:**

The online version contains supplementary material available at 10.1038/s41598-026-42340-2.

## Introduction

Rapeseed is an important economic crop primarily used for edible oil production, while also serving various other purposes. It can be used as green manure^1^, raw material for high-performance biodegradable plastic films^2^, and quality poultry feed^3^. In food applications, rapeseed combined with biscuits can create anti-aging products^4^. Additionally, it can be processed into soft drinks^5^, and its deproteinized fiber residues serve as resources for mushroom cultivation and feed production^6^.

Multiple factors determine rapeseed oil content. Appropriate nitrogen application is crucial for increasing seed oil content^7^. Oil bodies (OBs), as essential organelles, show a significant positive correlation with oil content^8,9^. To enhance rapeseed yield, effective fertilization strategies are vital. Foliar fertilization can significantly increase yield^10,11^, and proper timing of fertilizer application is critical^12^. Combining chemical fertilizers with appropriate organic fertilizers effectively promotes rapeseed growth^13,14^. Rapeseed has high sulfur (S) requirements, making proper S fertilization particularly important^15^. Flexible fertilizer application rates can increase yield while reducing costs and greenhouse gas emissions^16^.

Research on the *IQD* gene family has garnered attention across various model and non-model organisms, including *Vitis vinifera L.*^17^, *Citrullus lanatus*^18^, *Zea mays L.*^19^, *Malus pumila Mill.*^20^, *Arabidopsis thaliana*, *Oryza sativa L.*^21^, *Solanum tuberosum L.*^22^, *Gossypium hirsutum L.*^23^, *Triticum aestivum L.*^24^, and *Glycine max (L.) Merr.*^25^. Studies have shown that *IQD* genes are crucial for maintaining plant cell morphology^26^. They associate with microtubules and potentially facilitate cellular RNA localization^27^. Recent research has revealed that *IQD*22 plays a vital role in plant hypoxia tolerance, enhancing plant resistance^28^. In tomatoes, *SlIQD*21a functions in modifying fruit shape during development^29,30^, while in peppers, *CaIQD*1 serves as a key regulator of fruit morphology^31^. Other studies suggest that the *IQD* family represents the largest group of calmodulin-interacting proteins^32,33^. The IQ domain mediates interactions between plant calmodulin and cyclic nucleotide-gated channels, modifying cellular and organ morphology through calcium (Ca^2+^) signaling^33,34^. It also influences organ shape through microtubule interactions^35–37^. In cotton, *GhIQD*10 overexpression results in shorter fibers^38^. In *Arabidopsis*, *IQD*5 has been identified as a novel regulator of leaf epidermal cell morphology^39^ and stabilizes cortical microtubules (MTs)^40^. *IQD*9 shows high expression during seed development and participates in cell wall polysaccharide biosynthesis^41^. In *Populus deltoides*, *PdIQD*10 functions in secondary cell wall biosynthesis^42^.

Several *Brassica* crops are known as heavy metal accumulators. Brown mustard, in particular, exhibits rapid growth and metal tolerance, making it a focus of numerous studies due to its excellent phytoremediation potential^43,44^. While zinc is an essential micronutrient for plants, excessive concentrations can be toxic. Brown mustard shows significantly less root damage compared to cabbage-type oilseed rape under zinc stress^45^. In this study, using *Arabidopsis IQD* family members as reference sequences, we identified 107 *BjIQD* genes from the brown mustard genome. We conducted comprehensive analyses of their phylogenetic relationships, gene structures, protein physicochemical properties, homology, selection pressure, promoter cis-acting elements, interacting proteins, GO enrichment, and tissue-specific expression. Promoter cis-element analysis and protein interaction prediction indicate that BjIQD proteins play a role in responses to abiotic stress, which was further confirmed by qRT-PCR experiments. *Additionally*, *IQD* genes exhibit conservation during genomic structural evolution and have undergone strong purifying selection. We also discussed *BjIQD* genes lacking critical domains, providing insights into the evolutionary dynamics of *IQD* genes in *Brassica juncea*.

## Methods

### Identification of *IQD* gene family members in *Brassica juncea*

The complete genome sequence and annotation files of *Brassica juncea* were obtained NCBI (https://www.ncbi.nlm.nih.gov/)^46^. The *Brassica juncea* variety used in this study is Sichuan Huangzi, and the genome assembly version is ASM1870372v1. IQD protein sequences of *Arabidopsis thaliana* were retrieved from the *Arabidopsis* Information Resource (http://www.arabidopsis.org), based on these protein sequences, we identified 33 *AtIQD* members. The Hidden Markov Model (HMM) for IQD (PF00612) was obtained from the PFAM(https://www.ebi.ac.uk/interpro/entry/pfam/#table)^47^. *BjIQD* family members were identified through both BLAST (e-value ≤ 1e-5) alignment using *Arabidopsis* IQD protein sequences and HMM (e-value ≤ 0.01) seed model screening, yielding 122 and 200 members, respectively. The final *BjIQD* family members were determined after removing redundant genes from these results.

### Multiple sequence alignment and evolutionary analysis

MUSCLE in MEGA11 software was used for multiple sequence alignment, and the Maximum Likelihood (ML) method was employed for phylogenetic tree construction^48^. First, *Arabidopsis IQD* (*AtIQD*) family members were screened, with protein sequences and annotation files obtained from Ensembl Plants (http://plants.ensembl.org/index.html)^49^. The protein sequences of the *BjIQD* and *AtIQD* gene families were then combined, and phylogenetic trees were constructed using MEGA11 with a Bootstrap parameter set to 1000.

## Analysis of IQD conserved motifs and gene structure

The MEME online tool (https://meme-suite.org/meme/tools/meme)^50^ was used to predict conserved motifs in identified *BjIQD* members, with motif number set to 10 and other parameters at default values. Exon-intron analysis was performed using TBtools based on *Brassica juncea* annotation files and *BjIQD* member information. Finally, gene structure, conserved motifs, phylogenetic tree, and conserved domain analyses were visualized together.

### Chromosomal distribution and homology analysis of the *BjIQD* genes

To map *BjIQD* gene members to specific chromosomal locations, we analyzed and visualized their chromosomal positions using TBtools based on mustard annotation files. We performed intraspecific collinearity analysis for *Brassica juncea* and interspecific collinearity analysis between *Arabidopsis* and *B. juncea*, *Brassica rapa* and *B. juncea*, and *Brassica oleracea* and *B. juncea*. The protein sequences and annotation files for *B. rapa* and *B. oleracea* were obtained from the BRAD database (http://brassicadb.cn/#/)^51^.

### Physicochemical properties and subcellular localization of the *BjIQD* genes

We analyzed the physicochemical properties of the *BjIQD* family members using Expasy (https://web.expasy.org/protparam/)^52^, including predictions of amino acid count, molecular weight, and isoelectric point. The subcellular localization of the *BjIQD* members was predicted using the online tool WoLF PSORT (https://wolfpsort.hgc.jp/)^53^.

### Analysis of Cis-acting elements in promoters and gene selective pressure

We extracted 2000 bp upstream sequences from the start codons of the *BjIQD* genes from the *B. juncea* genome for cis-acting element analysis. These sequences were submitted to the PlantCARE(http://bioinformatics.psb.ugent.be/webtools/plantcare/html/)^54^ for identification of cis-acting elements, and results were visualized using TBtools. Using *Arabidopsis IQD* family genes as references, we conducted selection pressure analysis on *BjIQD* gene members using TBtools^55^, excluding sequences with effective continuous base lengths less than 3 after alignment.

#### Protein-protein interaction network prediction

To analyze BjIQD protein interactions, we used *Arabidopsis* as a model organism to map homologous proteins in mustard. Protein interactions were predicted using STRING (https://cn.string-db.org/)^56^and visualization using Cytoscape.

## GO enrichment analysis

To analyze the pathway coupling mechanisms of the *BjIQD* genes, based on the IQD protein sequences identified in *Brassica juncea*, we annotated *B. juncea* gene functions using the online platform EggNOG-mapper (http://eggnog-mapper.embl.de/)^57^. GO enrichment analysis was performed using TBtools based on the annotated files obtained.

### Tissue-specific expression analysis of the *BjIQD* family genes

In this study, we utilized RNA-seq transcriptome datasets retrieved from NCBI database, including root (SRR11787772), stem (SRR117807777), leaf (SRR11789776), bud (SRR11777782), seed (SRR11777781), and seed coat (SRR807368). After data analysis, heat maps of Log2(FPKM + 1) values were generated using the online tool ChiPlot (https://www.chiplot.online/).

### qRT-PCR analysis of the *BjIQD* family genes

When mustard-type rapeseed reached the bolting stage, stress treatment was applied. Root and leaf samples were collected after 24 h of stress treatment and ground in liquid nitrogen for RNA extraction. Six genes from the *BjIQD* gene family were selected for real-time fluorescence quantitative PCR analysis. Primers were designed using Primer5.0 software, with Actin as the internal reference gene. Primer sequences are provided in Table S6. The first strand cDNA synthesis was performed using BeyoRT™ II cDNA Kit (Beyotime Biotechnology, Shanghai, China). qRT-PCR analysis of the *BjIQD* genes was conducted on CFX96™ Real-Time System (Bio-Rad Laboratories, Hercules, California, USA) using SYBR Green qPCR Master Mix Kit (Sangon Biotech (Shanghai) Co., Ltd., Shanghai, China). The reaction parameters were set as: pre-denaturation at 95℃ for 2 min, followed by 40 cycles of denaturation at 95℃ for 10 s, annealing and extension at 60℃ for 30 s, and finally measuring the melting curve from 60℃ to 95℃. Three biological replicates and three technical replicates were set for each tissue, and the 2^-ΔΔCt method was used to calculate expression levels^58^.

## Results

### *BjIQD* family member identification and characterization

We identified 107 *BjIQD* members through BLAST alignment using *Arabidopsis* IQD protein amino acid sequences and HMM methodology. The *IQD* gene family primarily contains IQ and DUF4005 domains. In our results, some members contained both domains, while others contained only one domain, and some members lacked either or both domains. To study the evolutionary selection of the *IQD* gene family, we retained all identified results for comprehensive analysis, considering these genes essential for research purposes.

Physicochemical properties and subcellular localization analysis were conducted on 107 identified *BjIQD* gene members (Table S1). The average length of BjIQD proteins is 497 amino acids (aa), whereas that of *Arabidopsis* IQD proteins is 442 aa, with molecular weights ranging from 11.48 (*BjuB02g63340S*) to 192.3 (*BjuA06g28870S*) kDa. Their isoelectric points vary from 5.34 (*BjuA08g24640S*) to 12.73 (*BjuA03g16890S*). Most BjIQD proteins are basic, with only five acidic proteins (*BjuA08g24640S*,* BjuB04g25360S*,* BjuB03g51860S*,* BjuA06g28870S*,* BjuB04g23570S*). The instability index ranges from 37.53 (*BjuB03g16810S*) to 83.58 (*BjuA10g00670S*). With an index above 40 indicating instability, 97.2% of the BjIQD proteins are classified as unstable, while only three (*BjuB03g16810S*,* BjuA07g32910S*,* BjuB01g42220S*) are stable. The hydropathy index ranges from − 1.056 to -0.05, with all values below 0, indicating hydrophilic properties. Subcellular localization analysis shows most proteins are located in the nucleus (68.22%) and chloroplast (18.69%), with fewer in mitochondria (8.41%), plasma membrane (1.87%), cytosol (0.93%), endoplasmic reticulum (0.93%), and extracellular space (0.93%). These findings suggest functional diversity and synergy within the *BjIQD* gene family.

### Phylogenetic analysis of the *BjIQD* gene family

**Fig. 1:**
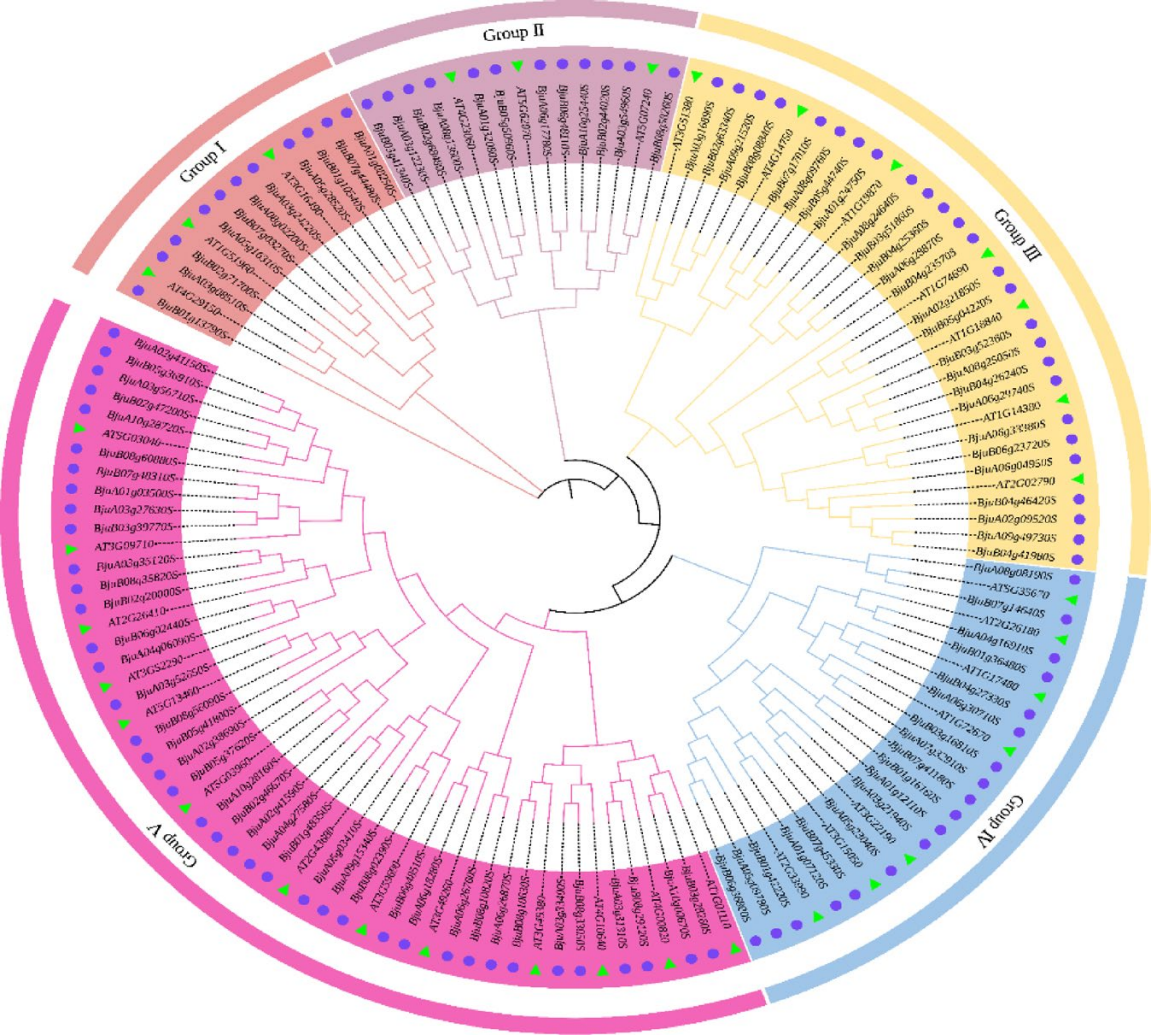
Phylogenetic tree of the *IQD* gene family in *Arabidopsis* and *Brassica juncea*. Blue circles represent *BjIQD* family members, and green triangles represent *AtIQD* family members.

To better understand *BjIQD* evolution, we constructed a phylogenetic tree using the Maximum Likelihood method, comparing BjIQD with AtIQD protein sequences. The results (Fig. 1) show five subfamilies based on *Arabidopsis* homologs. Group V contains the most IQD proteins with 40 *BjIQD* and 13 *AtIQD* genes, while Group I has the fewest with 14 members. Additionally, while *BjuB02g46670S* and *BjuA10g28160S* cluster together, *BjuB02g46670S* lacks any domains, whereas *BjuA10g28160S* contains both critical *IQD* family domains: IQ and DUF4005. Similarly, *BjuA01g07120S* and *BjuB07g45330S* lack domains yet share recent divergence with their *Arabidopsis* homolog *AT3G15050*. A comparable pattern exists among *BjuB02g47200S*, *BjuA10g28720S*, and *AT5G03040*, where *BjIQD* genes maintain *Arabidopsis* homology despite lacking key domains. These findings suggest that some *BjIQD* family members have undergone domain loss or alternative splicing during evolution.

### Analysis of conserved motifs, domains and gene structure

**Fig. 2:**
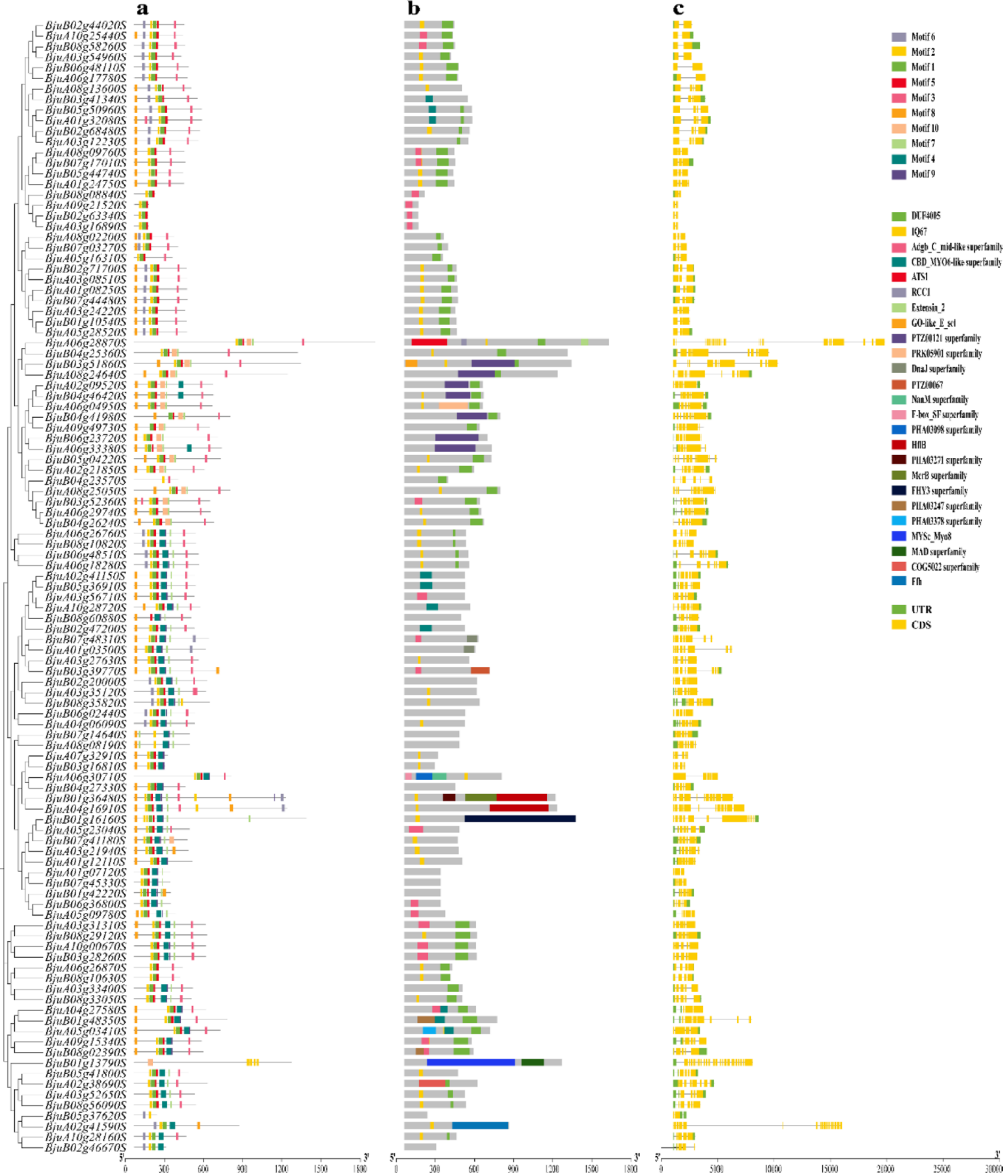
Evolutionary tree of the *BjIQD* genes and the analysis of conserved motifs, domains and gene structure. Note: *BjIQD* conserved motifs (**a**), conserved domains (**b**), gene structure (**c**).

To better understand the compositional diversity of the *BjIQD*, conserved motifs of *B. juncea IQD* family members were predicted using the MEME online tool. Ten conserved motifs were identified (Fig. 2a), These results suggest that motifs 1, 2 and 5 are characteristic motifs of the *BjIQD* genes. Analysis of the *BjIQD* gene structure (Fig. 2c) revealed that the minimum number of exons and introns was 2 and 1, respectively, while the maximum was 26 exons and 25 introns (*BjuA06g28870S*). Identification of the *BjIQD* conserved domains (Fig. 2b) showed that 30 *IQD*s lacked both IQ and DUF4005 domains, with 11 containing no domains at all, while the remaining IQD proteins contained at least one of either IQ or DUF4005 domains.

### Chromosomal distribution and homology analysis

Based on *B. juncea* genome annotation, the chromosomal locations of the *IQD* family genes were investigated (Fig. 5). In *B. juncea*, 107 *BjIQD* genes were distributed across 18 chromosomes. The A subgenome’s 10 chromosomes contained 54 *BjIQD* genes, while the B subgenome’s 8 chromosomes contained 53 *BjIQD* genes. Twelve *IQD* genes were located on chromosome A03, while chromosome A07 contained only one *IQD* gene.

**Fig. 3:**
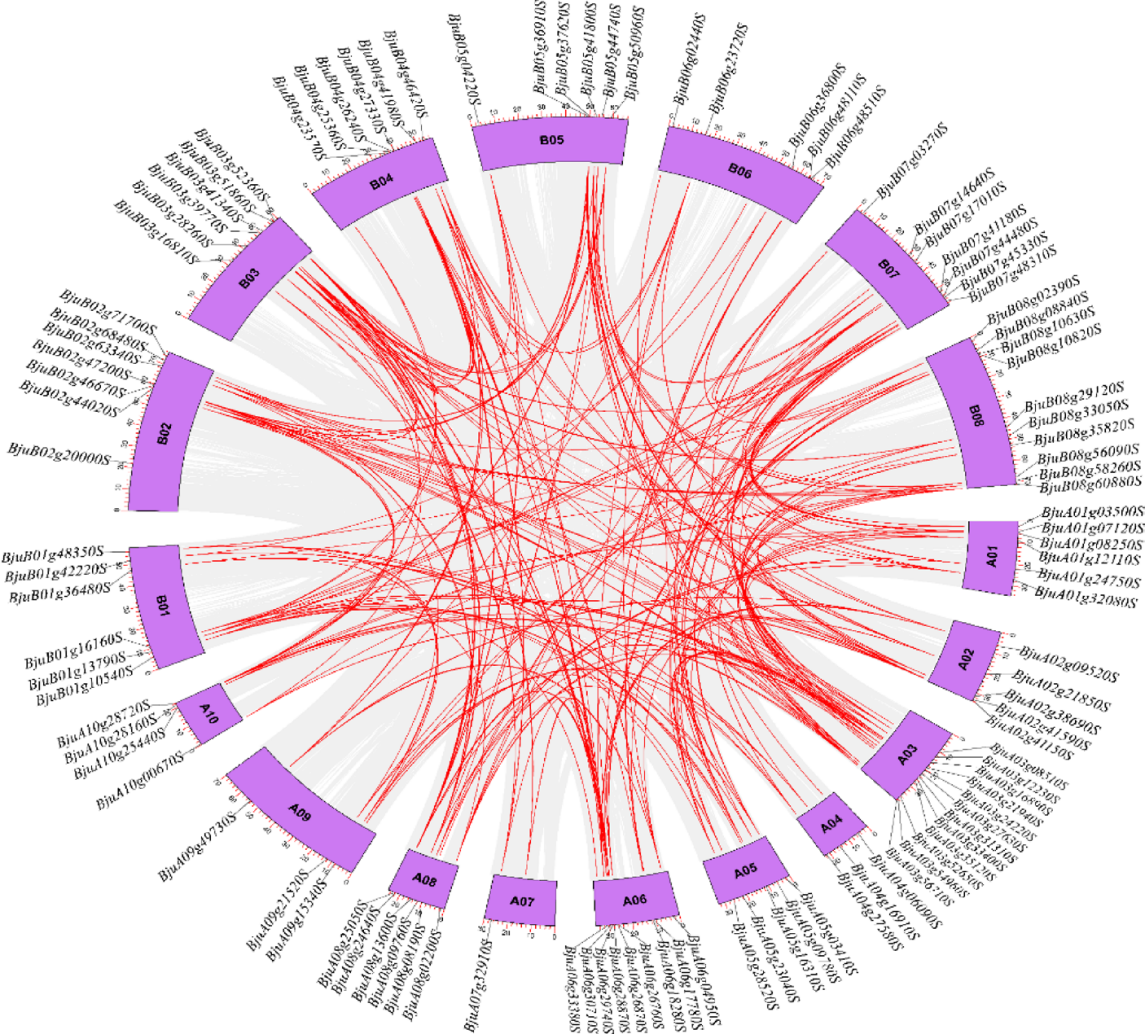
Duplication of the *BjIQD* genes on Brassica napus chromosomes.

**Fig. 4:**
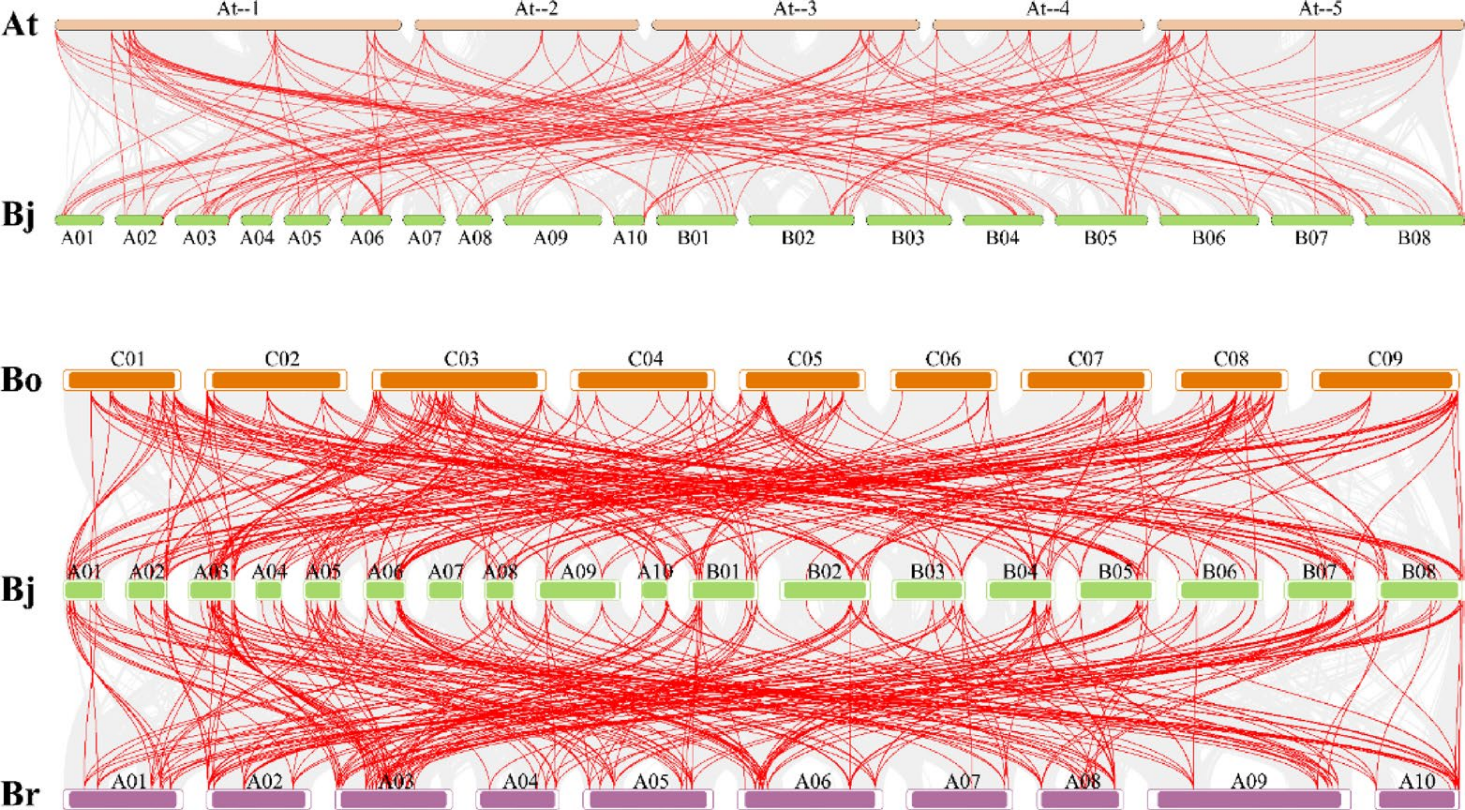
Synteny analysis of *IQD* genes between *Brassica napus* and *Arabidopsis*, *Brassica oleracea*, and *Brassica rapa*. Note: Gray lines in the background represent syntenic blocks between *Brassica napus* and other species, while red lines highlight homologous *IQD* gene pairs.

To determine the relationships among *BjIQD* members, we analyzed gene duplication events (Fig. 3). We identified 211 pairs of collinear genes, including 40, 131, and 40 homologous gene pairs between A-A, A-B, and B-B genomes, respectively. Analysis of the *BjIQD* gene duplication patterns revealed (Fig. S1) that segmental duplication was the primary mode of the *BjIQD* gene replication. To further evaluate the evolution and development of the *BjIQD* family, we compared the collinearity of *IQD* genes between *Brassica juncea* and *Arabidopsis*, *Brassica oleracea*, and *Brassica rapa* (Fig. 4). The results showed 148, 257, and 272 pairs of homologous genes between *B. juncea* and these three species, respectively. This suggests that the *BjIQD* gene family likely performs similar biological functions between *B. juncea* and *B. oleracea* and *B. rapa*.

#### Selection pressure analysis of the *BjIQD* members

To explore the evolutionary pressure on *BjIQD*, we calculated the non-synonymous mutation rate (Ka), synonymous mutation rate (Ks), and Ka/Ks values (Table S2). The results showed that all identified homologous *BjIQD* genes had Ka/Ks values less than 1, indicating that the *BjIQD* gene family underwent strong purifying selection during evolution.

**Fig. 5:**
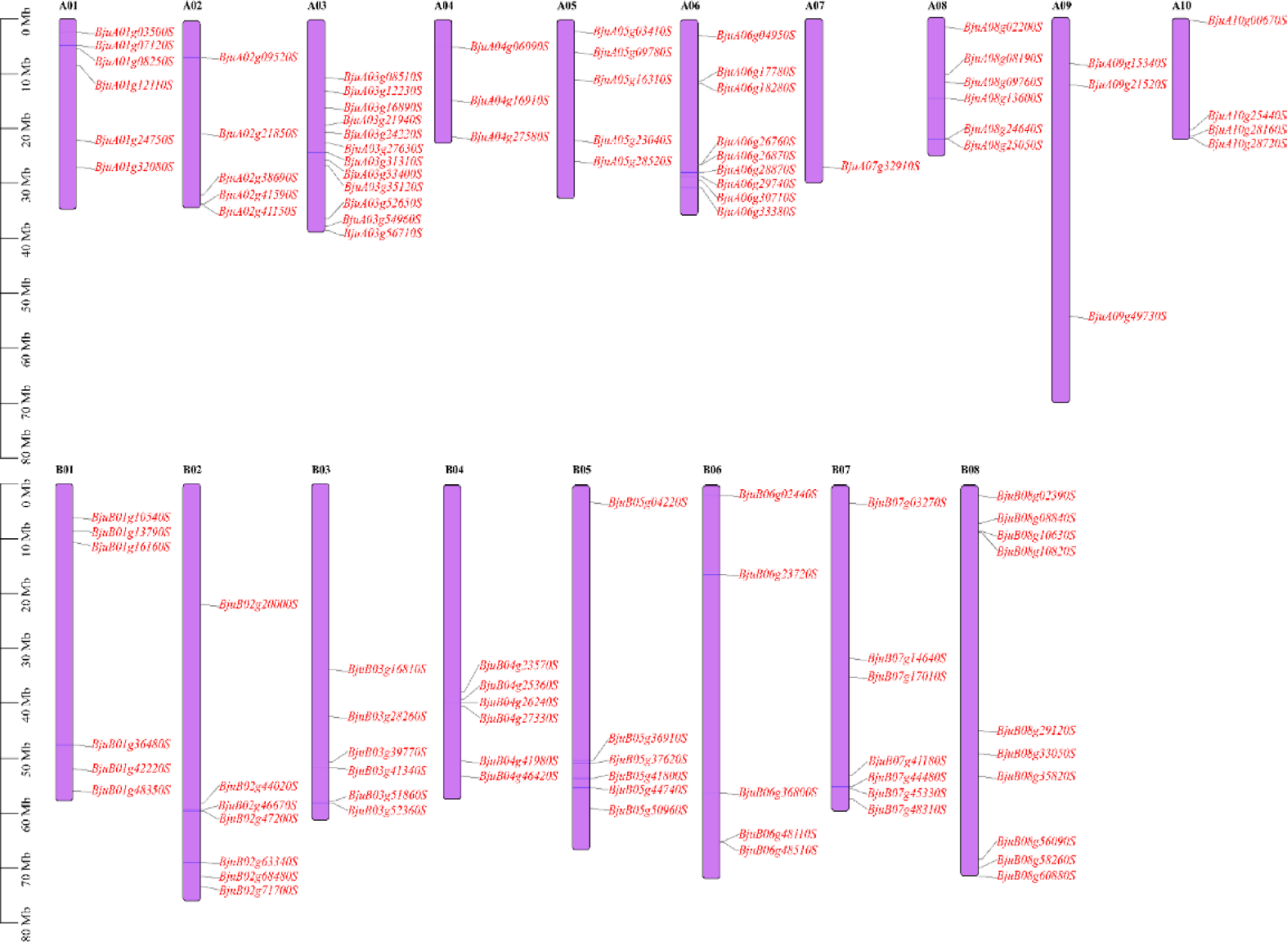
Distribution of the *IQD* family genes on *Brassica napus* chromosomes.

#### Analysis of promoter cis-acting elements

**Fig. 6:**
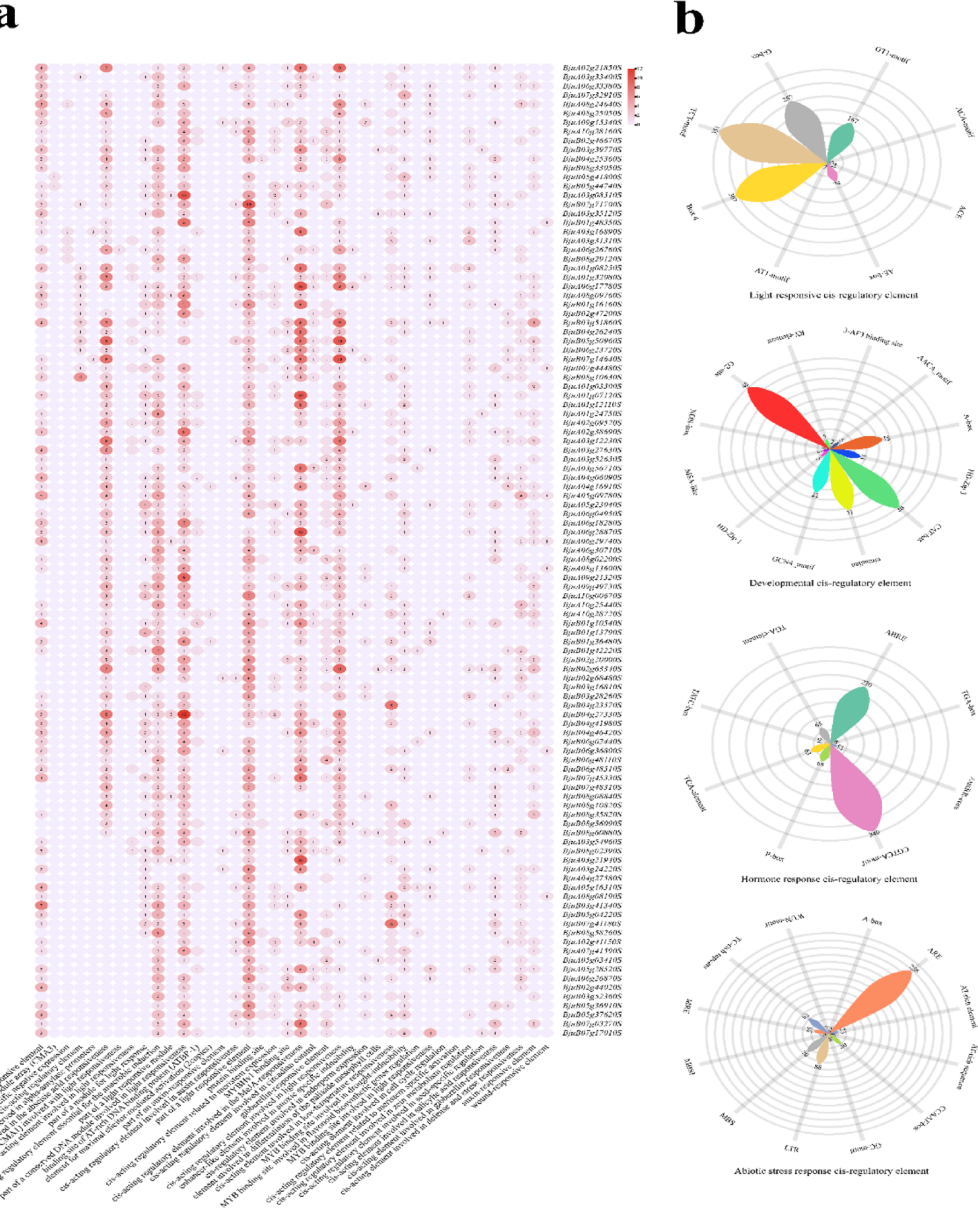
(**a**) Prediction results of cis-acting elements in *BjIQD* gene promoters. (**b**) Distribution of different categories of cis-acting elements in *BjIQD* promoter regions.

The analysis identified 40 types of cis-acting elements associated with *BjIQD* genes (Fig. 6a). These elements in the *BjIQD* promoters primarily function in light response, growth and development regulation, hormone response, and abiotic stress response. Based on these major functions, we classified these elements into four categories, as shown in Fig. 6b. Among the light-responsive elements, 1178 were identified, with Box 4, TCT-motif, G-box, and GT1-motif being particularly abundant. For growth and development regulation, 194 elements were identified, including elements related to corn protein metabolism (O2-site), meristem expression (CAT-box), circadian rhythm control (circadian), and endosperm cell differentiation (GCN4_motif). Hormone-responsive elements totaled 813, with methyl jasmonate response (CGTCA-motif) and abscisic acid response (ABRE) elements being most abundant, respectively. For abiotic stress response, 599 elements were predicted, dominated by anaerobic induction response (ARE) elements, followed by cold and drought response elements (LTR, MBS). Our analysis of the *BjIQD* members exhibiting domain loss reveals that these members retain functional characteristics typical of the *IQD* family. Integrating these findings, we conclude that *BjIQD* genes play important roles in plant growth, development, and stress responses.

#### Prediction of interacting proteins

**Fig. 7:**
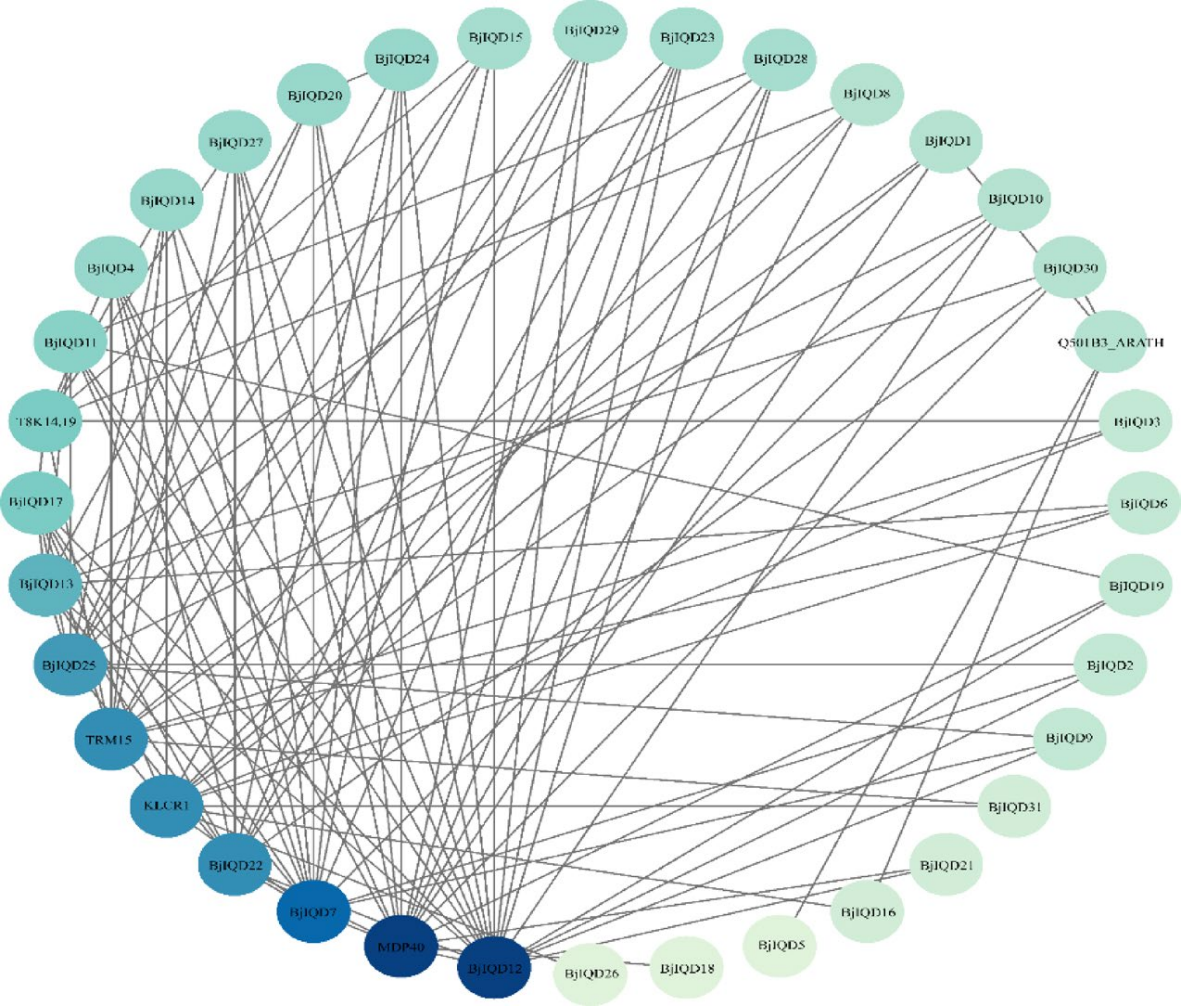
Predicted protein interaction network of the *BjIQD* gene family.

To analyze the functional predictions of the *BjIQD* gene family, we constructed a protein interaction network as shown in Fig. 7. For better analysis, the genes in the Fig. were renamed (Table S3). The results indicate that 31 BjIQD proteins interact with KLCR1, MDP40, Q501B3_ARATH, T8K14.19, and TRM15. Among these, the KLCR1 transcription factor enhances plant abiotic stress adaptation by coordinating immune responses, root secretions, and detoxification gene expression. The MDP40 transcription factor regulates plant morphogenesis by controlling microtubule skeleton dynamics in hypocotyl cell elongation. Q501B3_ARATH likely plays a pivotal role in hormone-mediated stress response and developmental regulation, while T8K14.19 may function in stress response and signal transduction regulation. TRM15 potentially participates in epigenetic regulation or hormone-mediated stress responses. These findings suggest that *BjIQD* family members have multiple regulatory pathways in plant stress response and signal transduction. Interestingly, we identified three genes without domains - *BjuA08g08190S*, *BjuB02g46670S*, and *BjuB07g45330S* - in this protein interaction network, these three genes correspond to *BjIQD*12, *BjIQD*17, and *BjIQD*25, with *BjIQD*12 showing the strongest interactions among all *BjIQD* members (Fig. 7). This indicates these genes likely perform functions similar to those of *IQD* family proteins.

### GO functional annotation analysis of the *BjIQD* genes

**Fig. 8:**
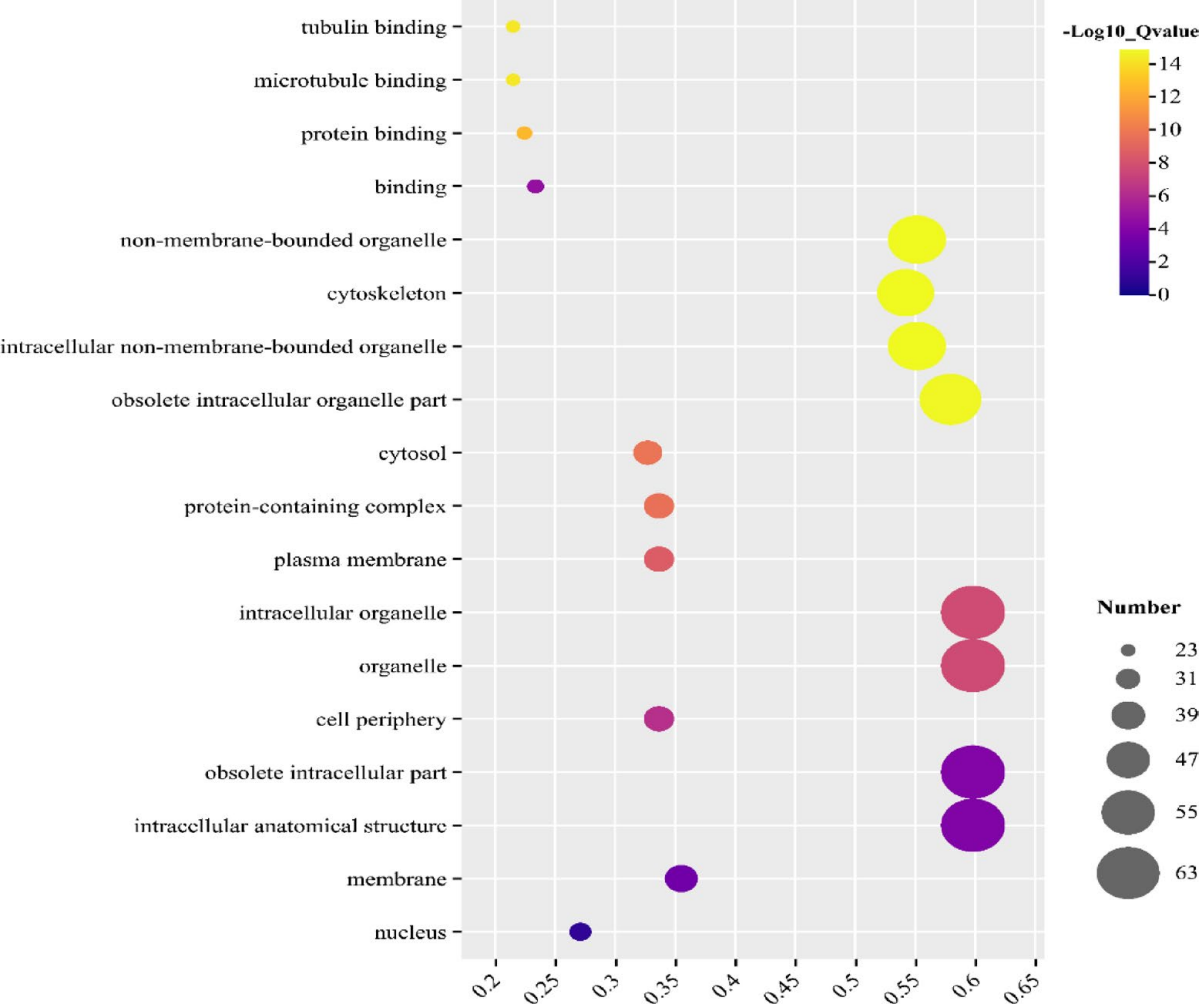
GO enrichment analysis results of the *BjIQD* genes. The vertical axis represents GO terms, and the horizontal axis shows gene proportion. Larger gene proportions indicate stronger enrichment, while dot size represents the number of genes in each GO term.

To systematically analyze the functional dimensions of the *BjIQD* genes, we performed functional enrichment analysis using Gene Ontology for structured interpretation. Results show that the *BjIQD* gene functions are categorized into molecular function and cellular component GO categories (Fig. 8, Table S4). Cellular component GO terms account for 77.78% of total entries, and molecular function annotations accounted for only 22.22%. In molecular function, “protein binding(GO:0005515)”, “tubulin binding(GO:0015631)”, and “microtubule binding(GO:0008017)” are the most enriched GO terms. In cellular components, “obsolete intracellular organelle part(GO:0044446)”, “non-membrane-bounded organelle(GO:0043228)”, “intracellular non-membrane-bounded organelle(GO:0043232)”, and “cytoskeleton(GO:0005856)” show high enrichment, while “intracellular organelle(GO:0043229)” and “organelle(GO:0043226)” are also notably enriched. These results indicate that the *BjIQD* gene functions are related to plant morphogenesis and stress response.

### Expression analysis of the *BjIQD* genes in different tissues and organs

**Fig. 9:**
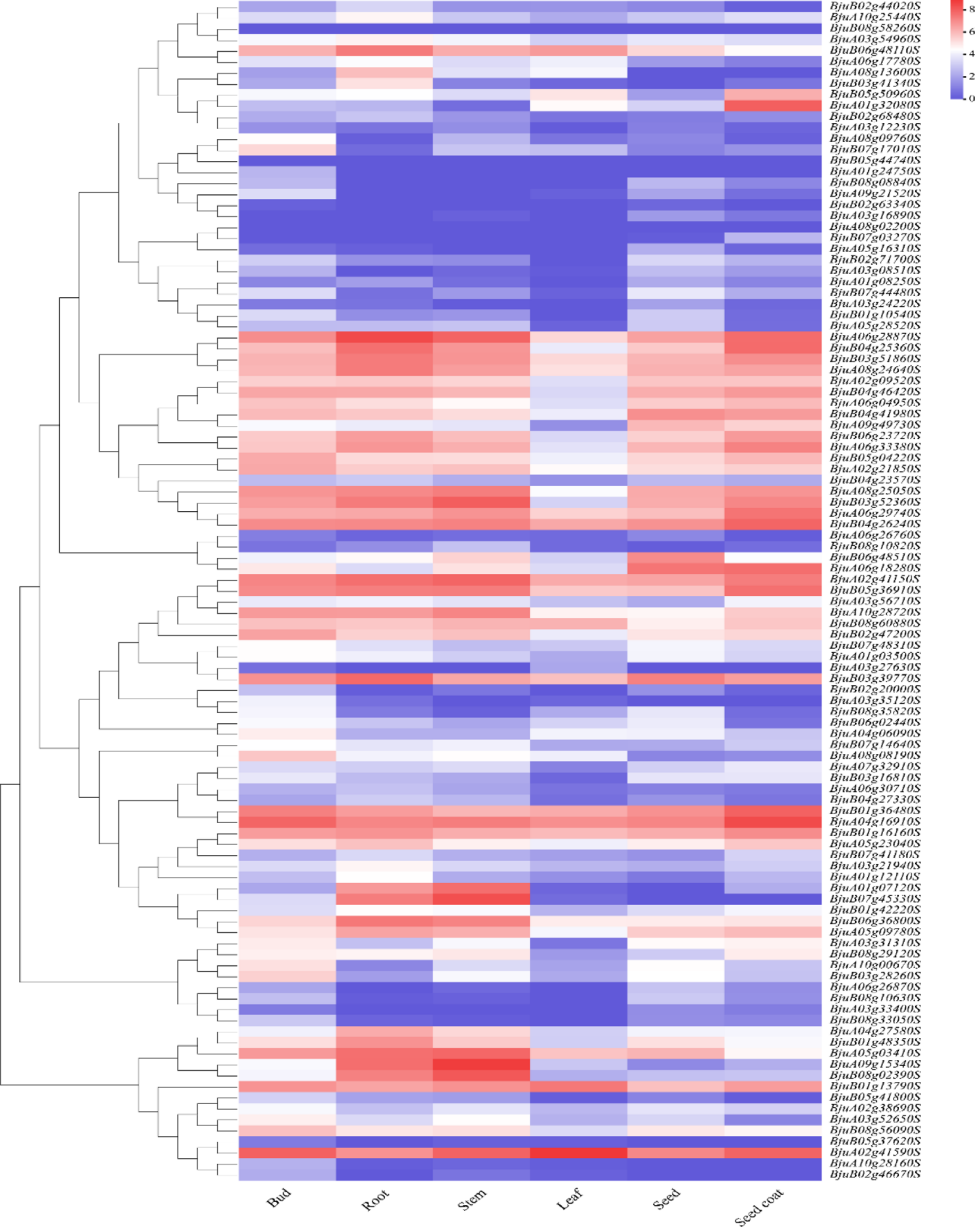
Tissue-specific expression analysis of *IQD* genes in *Brassica juncea*.

To better understand the tissue-specific expression patterns of the *BjIQD* genes, we analyzed their expression levels across different tissues using publicly available RNA-seq data (Table S5). The analyzed tissues included root, stem, leaf, bud, seed, and seed coat, with results shown in Fig. 9. The analysis revealed that most *BjIQD* genes exhibited high expression in at least two organs, with the most significant expression levels in stems and roots, followed by seed coat, while leaves showed the lowest expression. Among all *BjIQD* genes, *BjuA02g41590S* demonstrated high expression in leaves and maintained relatively high expression in other tissues. *BjuA06g28870S* showed high expression in roots and maintained elevated expression across all tissues except leaves. Meanwhile, four genes - *BjuA09g15340S*, *BjuB07g45330S*, *BjuA01g07120S*, and *BjuB08g02390S* - exhibited high expression exclusively in roots and stems, with minimal expression in other tissues. Interestingly, our analysis of the *BjIQD* genes with missing or incomplete important domains revealed varying expression levels across different tissues, differing only in expression intensity. These results indicate tissue-specific expression patterns of the *BjIQD* genes.

### qRT-PCR analysis of the *BjIQD* gene expression patterns under abiotic stress

**Fig. 10:**
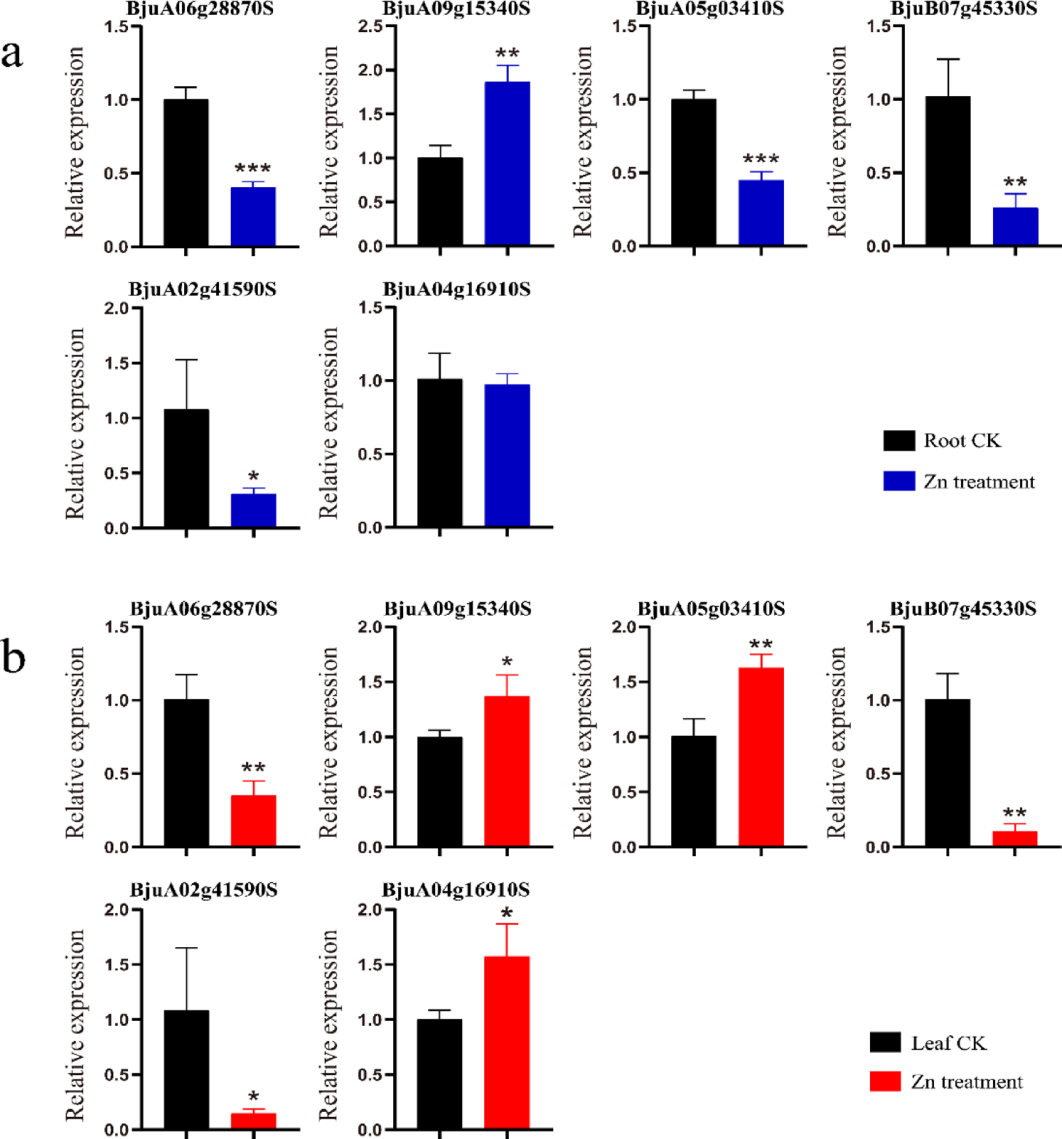
(**a**) Expression levels of the *BjIQD* genes in *B. juncea* roots under Zn stress treatment. (**b**) Expression levels of the *BjIQD* genes in *B. juncea* leaves under Zn stress treatment.

To investigate the effects of abiotic stress on *BjIQD* gene expression, we used Zn as a stimulus and studied *BjIQD* expression in both roots and leaves. Under Zn treatment, six *BjIQD* genes showed distinct expression patterns in roots and leaves (Fig. 10). *BjuA06g28870S*, *BjuB07g45330S*, and *BjuA02g41590S* exhibited significant downregulation in both roots and leaves. *BjuA05g03410S* and *BjuA04g16910S* showed downregulation in roots, but upregulation in leaves. *BjuA09g15340S* demonstrated notable upregulation in both roots and leaves.

## Discussion

IQD proteins play crucial roles in plant growth regulation and environmental stress response. Multiple studies have shown that IQD proteins interact with microtubules, affecting cell morphology and development^27,36^. They also participate in calcium ion signaling to regulate plant growth^33^ and contribute to oxidative stress tolerance^28^. Previous studies have reported 33 *IQD* genes in *Arabidopsis*^21^, and varying numbers in other species: 29 in *Oryza sativa L.*^21^, 49 in *Vitis vinifera L.*^17^, 35 in *Citrullus lanatus*^18^, 26 in *Zea mays L.*^19^, 42 in *Malus pumila Mill.*^20^, 23 in *Solanum tuberosum L.*^22^, 102 in *Gossypium hirsutum L.*^23^, 125 in *Triticum aestivum L.*^24^, and 67 in *Glycine max (L.) Merr.*^25^. While *IQD* family genes in *Brassica juncea* remained unreported, our study identified 107 *IQD* genes, representing a relatively high number compared to previously reported plants. Most BjIQD proteins are basic and unstable, with hydrophilicity indices below 0, indicating their hydrophilic nature. Subcellular localization predictions show that while most IQD proteins localize to the nucleus, some distribute to chloroplasts, mitochondria, and plasma membranes, suggesting functional diversity and synergy in IQD protein activities.

Phylogenetic analysis revealed that *Brassica juncea IQD* genes are divided into 5 subfamilies. The number of *IQD* subfamilies varies among different plants - for example, *Zea mays L.* and *Solanum tuberosum L.* have 4 subfamilies^19,22^, while *Malus pumila Mill.* and *Glycine max (L.) Merr.* have 5^20,25^. According to Fig. 2, motif 1, motif 2, and motif 5 represent characteristic motifs of the *BjIQD* genes, not all 107 *IQD* members contain these three motifs, possibly due to functional differentiation of IQD proteins during evolutionary adaptation. *BjIQD* genes are distributed across 18 chromosomes, with similar numbers on A and B subgenomes. The number of homologous gene pairs between A-A and B-B groups is equal. Segmental duplication appears to be the main duplication mode for *BjIQD* genes, the predominant replication pattern of IQD genes in other plant species is similar^23,59,60^. Combined with Ka/Ks ratios less than 1, this suggests the *IQD* gene family is conserved in genome structural evolution and has undergone strong purifying selection^61^. The cis-elements in *BjIQD* promoters can be classified into four categories: light response, growth and development regulation, hormone response, and abiotic stress response. These elements validate the basic roles of *IQD* genes in regulating plant growth, development and stress responses. For example, hormone-responsive elements CGTCA-motif and ABRE are involved in methyl jasmonate and abscisic acid responses. Abiotic stress elements ARE, LTR and MBS participate in anaerobic induction, cold and drought responses, respectively. The predicted protein interaction network further confirms their functions in plants. Among these, transcription factors KLCR1, Q501B3_ARATH and TRM15 enhance plant adaptation to abiotic stresses in different ways. The MDP40 transcription factor affects plant morphogenesis by regulating microtubules. T8K14.19 plays a regulatory role in signal transduction. These transcription factor functions align with previous reports on the *IQD* family. If both promoter cis-element analysis and protein–protein interaction networks include indicators related to abiotic stress, this would serve as strong evidence supporting the involvement of the BjIQD gene family in abiotic stress responses. GO functional annotation analysis categorized *BjIQD* genes into molecular function and cellular component categories, with cellular components containing the most entries. Among these entries, microtubule-related GO terms (GO:0015631, GO:0008017) were most prevalent, further confirming the biological functions of the *IQD* family.

*BjIQD* family members primarily consist of two key domains - IQ and DUF4005. Besides the IQ domain, DUF4005 is also a microtubule-binding domain^62^. Some genes lack these domains, but we hypothesize they may function through unknown domains. Therefore, we retained these genes in our *BjIQD* family analysis rather than removing them as pseudogenes. In phylogenetic analysis, genes lacking any domains still clustered with those containing key domains. When not in the same cluster, homologous *Arabidopsis IQD* genes could be found among recently diverged genes. Gene structure analysis showed these genes contain at least one exon. Promoter cis-element analysis revealed these members possess *IQD* family functional characteristics with corresponding elements. Protein interaction predictions identified three *IQD* family genes without domains, with one member showing the strongest interactions in the protein interaction network. These genes exhibit differential expression across various tissues. Quantitative real-time PCR (qRT-PCR) analysis of one representative gene showed relatively high expression levels, suggesting these genes are functionally active and may be expressed during specific developmental stages or under particular environmental conditions^60^. This phenomenon may result from gene simplification or alternative splicing during evolution.

RNA-Seq analysis revealed varying expression levels of the *BjIQD* genes across different tissues, with notably high expression in roots, stems, and seed coats. This indicates tissue-specific expression patterns of the *BjIQD* genes. Zinc stress affects plant metabolic processes and inhibits plant growth and development^63^. In this study, zinc was used as an abiotic stress stimulus. qRT-PCR analysis showed differential expression of these *BjIQD* genes in both roots and leaves. Since *Brassica juncea* is an allotetraploid species with an AABB genome, primer design must account for homologous gene copies. Primer5.0 designs primers based on physicochemical parameters such as length, melting temperature, and GC content. However, specific primer design requires additional biological specificity criteria—including uniqueness, intraspecific and interspecific specificity—which Primer5.0 cannot adequately evaluate in allopolyploid genomes. To confirm primer specificity, rigorous experimental validation—such as PCR amplification—is necessary, in addition to detailed bioinformatics analysis^64,65^. Returning to our experimental results, specifically, three genes exhibited significant downregulation; two genes showed downregulation in roots, but upregulation in leaves; and one gene demonstrated clear upregulation in both roots and leaves. These findings suggest functional differentiation among *BjIQD* family members, with both positive and negative feedback regulation in plant modulation.

## Conclusions

This study conducted bioinformatics analysis of *IQD* genes in *Brassica juncea*, identifying 107 *IQD* genes. All BjIQD proteins exhibited hydrophilic properties. Fragment duplication emerged as the primary replication mode for *BjIQD* genes, which underwent strong purifying selection during evolution. Promoter cis-element prediction and protein interaction analysis revealed functional diversity within the *IQD* family. GO functional annotation showed enrichment in microtubule-related GO terms. RNA-Seq and qRT-PCR analyses confirmed tissue-specific expression of the *BjIQD* genes. Additionally, we observed that genes lacking relevant domains exhibit functions similar to *IQD* family proteins. This phenomenon may result from gene simplification during evolution or alternative splicing. These findings provide new insights for future research on the *IQD* family.

## Supplementary Information

Below is the link to the electronic supplementary material.

Supplementary Material 1

Supplementary Material 2

Supplementary Material 3

Supplementary Material 4

Supplementary Material 5

Supplementary Material 6

Supplementary Material 7

## Data Availability

All data generated or analyzed in this study are included in this article or supplementary materials. Sequences of *Brassica juncea* , *Brassica rapa* , and *Brassica oleracea* used in this study were obtained from the BRAD database (http://brassicadb.cn/#/) and NCBI (https://www.ncbi.nlm.nih.gov/). Arabidopsis thaliana sequences were obtained from Ensembl Plants website (http://plants.ensembl.org/index.html), and Arabidopsis thaliana IQD protein sequences were obtained from The Arabidopsis Information Resource (http://www.arabidopsis.org). The HMM model was obtained from PFAM (https://www.ebi.ac.uk/interpro/entry/pfam/#table) using number “PF00612”. Transcriptome data used in this study were obtained from NCBI database (https://www.ncbi.nlm.nih.gov/) under accession numbers SRR11787772, SRR117807777, SRR11789776, SRR11777782, SRR11777781, and SRR807368.
