# Supplementary material for: Structure, evolution, phylogeny, and analysis of domain-deficient genes in the IQD gene family of Brassica juncea
Source: Sci Rep. 2026 Mar 2;16:11773. doi: 10.1038/s41598-026-42340-2 (PMC13065986; doi:10.1038/s41598-026-42340-2)
Supplement: Supplementary file 2 — Supplementary Material 2 [file 41598_2026_42340_MOESM2_ESM.pdf]

# Table S1 Characterization of IQD proteins

| Gene name | Number of Amino Acid | Molecular Weight(kDa) | Isoelectric point | Instability Index | Grand Average of Hydropathicity | Subcellular localization |
|---|---|---|---|---|---|---|
| BjuA06g04950S | 576 | 62.80 | 9.61 | 49.19 | -0.824 | Nucleus |
| BjuB05g04220S | 638 | 70.78 | 9.46 | 54.82 | -0.536 | Plasma membrane |
| BjuB05g41800S | 394 | 45.22 | 10 | 68.6 | -0.894 | Nucleus |
| BjuB07g48310S | 546 | 60.63 | 10.32 | 62.66 | -0.831 | Nucleus |
| BjuA01g24750S | 366 | 41.28 | 9.78 | 68.63 | -0.792 | Nucleus |
| BjuB07g14640S | 407 | 46.19 | 9.37 | 69.95 | -0.686 | Chloroplast |
| BjuA07g32910S | 245 | 27.34 | 10.41 | 38.22 | -0.538 | Nucleus |
| BjuA04g27580S | 528 | 58.29 | 10.53 | 69.58 | -0.891 | Nucleus |
| BjuB02g44020S | 368 | 41.49 | 10.53 | 76.68 | -0.91 | Nucleus |
| BjuA01g08250S | 389 | 43.51 | 10.12 | 48.34 | -0.68 | Nucleus |
| BjuA06g26760S | 451 | 50.13 | 9.71 | 50.92 | -0.824 | Nucleus |
| BjuA08g08190S | 406 | 46.17 | 8.81 | 75.22 | -0.972 | Nucleus |
| BjuB06g02440S | 447 | 49.37 | 10.65 | 62.63 | -0.792 | Nucleus |
| BjuA08g02200S | 290 | 32.88 | 10.4 | 50.59 | -0.661 | Mitochondrion |
| BjuB08g58260S | 370 | 41.70 | 10.41 | 68.91 | -0.864 | Chloroplast |
| BjuB06g48110S | 399 | 44.21 | 10.47 | 75.52 | -0.875 | Chloroplast |
| BjuB05g50960S | 497 | 54.80 | 10.24 | 77.39 | -0.791 | Nucleus |
| BjuA03g31310S | 525 | 58.62 | 10.33 | 82.2 | -0.966 | Nucleus |
| BjuA06g26870S | 352 | 40.13 | 10.27 | 53.99 | -0.878 | Nucleus |
| BjuB01g16160S | 1268 | 143.85 | 9.06 | 53.8 | -0.605 | Nucleus |
| BjuA02g09520S | 578 | 63.28 | 9.53 | 49.9 | -0.897 | Nucleus |
| BjuA06g29740S | 564 | 62.46 | 9.17 | 54.91 | -0.77 | Nucleus |
| BjuA03g27630S | 474 | 53.13 | 10.52 | 64.49 | -1.026 | Nucleus |
| BjuB02g68480S | 481 | 53.45 | 10.06 | 73.6 | -0.797 | Nucleus |
| BjuB05g37620S | 167 | 18.76 | 10.39 | 50.25 | -0.186 | Nucleus |
| BjuB03g39770S | 627 | 69.81 | 10.47 | 56.83 | -0.9 | Cytosol |
| BjuB01g13790S | 1160 | 130.88 | 8.54 | 49.88 | -0.492 | Nucleus |
| BjuB02g71700S | 384 | 41.36 | 10.7 | 55.76 | -0.368 | Chloroplast |
| BjuA09g15340S | 495 | 55.99 | 11.06 | 66.83 | -0.95 | Nucleus |
| BjuA03g33400S | 431 | 49.13 | 9.97 | 67.77 | -0.802 | Nucleus |
| BjuA02g41150S | 446 | 48.78 | 10.62 | 63.05 | -0.885 | Nucleus |
| BjuB08g02390S | 507 | 57.05 | 11.07 | 67.17 | -0.975 | Nucleus |
| BjuA02g38690S | 538 | 60.84 | 9.86 | 74.01 | -0.893 | Nucleus |
| BjuB06g48510S | 473 | 52.5 | 10.06 | 44.91 | -0.885 | Nucleus |
| BjuB01g10540S | 383 | 43.04 | 10.21 | 57.76 | -0.675 | Nucleus |
| BjuA08g09760S | 368 | 41.47 | 9.9 | 79.92 | -0.847 | Nucleus |
| BjuB06g23720S | 613 | 66.83 | 9.32 | 50.56 | -0.813 | Nucleus |
| BjuA06g33380S | 645 | 70.6 | 8.9 | 54.69 | -0.768 | Nucleus |
| BjuA10g28160S | 382 | 43.88 | 10.54 | 66.92 | -0.727 | Nucleus |
| BjuA01g03500S | 523 | 58.13 | 10.55 | 58.42 | -0.858 | Nucleus |

| | | | | | | |
|---|---|---|---|---|---|---|
| BjuB06g36800S | 265 | 29.9 | 10.54 | 40.73 | -0.773 | Nucleus |
| BjuA08g13600S | 420 | 46.47 | 10.39 | 73.72 | -0.71 | Chloroplast |
| BjuB08g10630S | 338 | 38.48 | 10.4 | 53.54 | -0.833 | Chloroplast |
| BjuA08g25050S | 706 | 78.05 | 9.25 | 55.12 | -0.626 | Endoplasmic reticulum |
| BjuA03g35120S | 529 | 58.52 | 10.21 | 60.4 | -0.919 | Nucleus |
| BjuA03g24220S | 373 | 41.93 | 10.06 | 53.34 | -0.734 | Nucleus |
| BjuA01g12110S | 426 | 47.53 | 10.03 | 59.76 | -0.589 | Chloroplast |
| BjuB03g52360S | 557 | 61.17 | 9.36 | 58.83 | -0.764 | Chloroplast |
| BjuB01g48350S | 682 | 76.06 | 10.11 | 65.15 | -0.731 | Nucleus |
| BjuB05g44740S | 358 | 40.69 | 9.96 | 66.97 | -0.766 | Nucleus |
| BjuA02g21850S | 516 | 56.87 | 9.63 | 58.62 | -0.78 | Nucleus |
| BjuA01g07120S | 264 | 30.28 | 10.31 | 49.55 | -0.721 | Nucleus |
| BjuB01g36480S | 1116 | 122.34 | 9.9 | 48.05 | -0.423 | Nucleus |
| BjuA02g41590S | 772 | 86.5 | 10 | 44.83 | -0.305 | Chloroplast |
| BjuB07g17010S | 376 | 42.31 | 9.86 | 82.73 | -0.878 | Nucleus |
| BjuA04g16910S | 1126 | 122.85 | 9.83 | 48.84 | -0.394 | Nucleus |
| BjuB04g23570S | 322 | 35.46 | 6.92 | 42.57 | -0.457 | Chloroplast |
| BjuA06g28870S | 1776 | 192.3 | 6.54 | 46.33 | -0.66 | Nucleus |
| BjuA01g32080S | 498 | 54.91 | 10.25 | 76.63 | -0.762 | Nucleus |
| BjuB02g20000S | 534 | 58.43 | 10.23 | 50.45 | -0.896 | Extracellular |
| BjuA03g56710S | 443 | 48.59 | 10.6 | 66.16 | -0.893 | Nucleus |
| BjuA06g30710S | 716 | 80.91 | 9.57 | 52.05 | -0.51 | Nucleus |
| BjuB08g33050S | 424 | 48.51 | 9.93 | 69.87 | -0.8 | Nucleus |
| BjuB01g42220S | 265 | 29.95 | 10.51 | 38.41 | -0.778 | Nucleus |
| BjuA03g21940S | 397 | 44.13 | 10.71 | 51.59 | -0.714 | Nucleus |
| BjuB07g44480S | 393 | 43.86 | 10.16 | 50.95 | -0.677 | Nucleus |
| BjuA10g00670S | 527 | 59.28 | 10.32 | 83.58 | -1.038 | Nucleus |
| BjuB04g41980S | 707 | 77.04 | 8.93 | 44.62 | -0.607 | Nucleus |
| BjuB08g35820S | 555 | 61.21 | 10.37 | 53.08 | -0.921 | Nucleus |
| BjuB03g41340S | 465 | 51.71 | 10.36 | 76.59 | -0.768 | Chloroplast |
| BjuA05g03410S | 632 | 69.6 | 11.57 | 77.97 | -0.963 | Nucleus |
| BjuB02g63340S | 100 | 11.48 | 12.58 | 78.83 | -0.121 | Mitochondrion |
| BjuA03g12230S | 471 | 52.59 | 10.08 | 69.62 | -0.801 | Nucleus |
| BjuA03g16890S | 103 | 11.81 | 12.73 | 78.83 | -0.088 | Mitochondrion |
| BjuB02g47200S | 444 | 48.7 | 10.44 | 59.25 | -0.875 | Nucleus |
| BjuB08g08840S | 150 | 17.1 | 12.13 | 70.57 | -0.127 | Chloroplast |
| BjuA05g16310S | 280 | 32.3 | 10.46 | 46.6 | -0.851 | Nucleus |
| BjuB05g36910S | 449 | 49.17 | 10.58 | 64.06 | -0.874 | Nucleus |
| BjuB03g51860S | 1230 | 134.33 | 6.14 | 51.38 | -0.71 | Chloroplast |
| BjuB08g60880S | 418 | 46.1 | 10.37 | 64.62 | -1.007 | Nucleus |
| BjuB03g28260S | 529 | 59.65 | 10.26 | 80.57 | -1.056 | Nucleus |
| BjuB04g25360S | 1205 | 130.25 | 5.4 | 49.34 | -0.676 | Nucleus |
| BjuB08g29120S | 535 | 59.74 | 10.18 | 82.37 | -1.017 | Chloroplast |
| BjuA10g28720S | 485 | 53.28 | 10.12 | 60.89 | -0.762 | Nucleus |

| | | | | | | |
|---|---|---|---|---|---|---|
| BjuA03g52650S | 446 | 50.85 | 10.16 | 68.04 | -0.931 | Nucleus |
| BjuA03g54960S | 343 | 38.58 | 10.41 | 71.16 | -0.8 | Chloroplast |
| BjuB07g41180S | 392 | 43.97 | 10.25 | 64.23 | -0.766 | Mitochondrion |
| BjuA09g49730S | 554 | 60.47 | 9.71 | 50.59 | -0.882 | Nucleus |
| BjuA06g17780S | 393 | 43.9 | 10.48 | 73.36 | -0.872 | Chloroplast |
| BjuB04g27330S | 375 | 41.64 | 10.52 | 63.24 | -0.693 | Nucleus |
| BjuB07g03270S | 322 | 36.53 | 10.36 | 49.82 | -0.66 | Mitochondrion |
| BjuA05g09780S | 300 | 33.84 | 10.66 | 42.19 | -0.575 | Chloroplast |
| BjuA09g21520S | 103 | 11.74 | 12.42 | 77.01 | -0.05 | Mitochondrion |
| BjuA05g23040S | 406 | 45.05 | 10.43 | 47.07 | -0.777 | Mitochondrion |
| BjuB02g46670S | 234 | 26.78 | 11.21 | 48.53 | -0.531 | Mitochondrion |
| BjuA06g18280S | 475 | 52.83 | 9.92 | 50.67 | -0.914 | Nucleus |
| BjuB04g46420S | 583 | 63.26 | 9.65 | 46.56 | -0.875 | Nucleus |
| BjuA03g08510S | 388 | 41.61 | 10.86 | 49.15 | -0.393 | Chloroplast |
| BjuB03g16810S | 224 | 24.94 | 10.45 | 37.53 | -0.601 | Nucleus |
| BjuA05g28520S | 387 | 43.61 | 10.16 | 56.42 | -0.685 | Nucleus |
| BjuA08g24640S | 1130 | 122.04 | 5.34 | 55.18 | -0.755 | Nucleus |
| BjuA04g06090S | 445 | 49.05 | 10.73 | 63.61 | -0.771 | Nucleus |
| BjuB07g45330S | 264 | 30.33 | 10.53 | 47.72 | -0.695 | Chloroplast |
| BjuA10g25440S | 354 | 39.69 | 10.7 | 69.57 | -0.848 | Mitochondrion |
| BjuB08g56090S | 452 | 51.62 | 10.1 | 69.46 | -0.969 | Nucleus |
| BjuB04g26240S | 586 | 65.03 | 8.77 | 54.98 | -0.633 | Plasma membrane |
| BjuB08g10820S | 452 | 50.21 | 9.68 | 48.67 | -0.857 | Chloroplast |
