# Supplementary material for: Structure, evolution, phylogeny, and analysis of domain-deficient genes in the IQD gene family of Brassica juncea
Source: Sci Rep. 2026 Mar 2;16:11773. doi: 10.1038/s41598-026-42340-2 (PMC13065986; doi:10.1038/s41598-026-42340-2)
Supplement: Supplementary file 3 — Supplementary Material 3 [file 41598_2026_42340_MOESM3_ESM.pdf]

Table S2 Selection Pressure Analysis of the IQD Members

Gene name in A. thaliana	Gene name in B. napus	Ka	Ks	Ka/Ks
AT1G14380.3	BjuA06g04950S	0.240731002	0.600517248	0.400872752
AT1G74690.1	BjuB05g04220S	0.09806645	0.320613103	0.305871623
AT5G13460.1	BjuB05g41800S	0.134450666	0.349499409	0.384694972
AT3G09700.1	BjuB07g48310S	0.070009639	0.353115217	0.198262878
AT4G14750.3	BjuA01g24750S	0.117147298	0.489083479	0.239524137
AT5G35670.1	BjuB07g14640S	0.134310722	0.433965973	0.309495976
AT1G72670.1	BjuA07g32910S	0.099430823	0.548678627	0.181218692
AT2G43680.1	BjuA04g27580S	0.131568881	0.37670269	0.34926451
AT5G62070.1	BjuB02g44020S	0.221259512	1.460251926	0.151521466
AT1G51960.1	BjuA01g08250S	0.265526854	1.062324466	0.249948921
AT3G49260.3	BjuA06g26760S	0.065778673	0.411763907	0.159748515
AT5G35670.1	BjuA08g08190S	0.108087118	0.437890142	0.246836152
AT3G52290.1	BjuB06g02440S	0.099408	0.407453413	0.243973904
AT1G51960.1	BjuA08g02200S	0.173965636	0.553779209	0.314142591
AT5G62070.1	BjuB08g58260S	0.286458305	1.195252065	0.23966351
AT5G07240.2	BjuB06g48110S	0.185805111	1.64698064	0.112815601
AT4G23060.1	BjuB05g50960S	0.102359372	0.457674602	0.223650978
AT1G01110.2	BjuA03g31310S	0.155016534	1.090398315	0.142165052
AT3G49380.1	BjuA06g26870S	0.130601979	0.420650354	0.310476332
AT3G22170.2	BjuB01g16160S	0.064542791	0.607437043	0.106254288
AT1G14380.3	BjuA02g09520S	0.249324498	0.653372964	0.381595982
AT1G18840.3	BjuA06g29740S	0.107137672	0.349932072	0.306167056
AT3G09710.2	BjuA03g27630S	0.166746202	0.432028329	0.385961268
AT4G23060.1	BjuB02g68480S	0.115606531	0.485952992	0.23789653
AT5G03960.3	BjuB05g37620S	0.245978911	0.616696337	0.39886553
AT3G09680.1	BjuB03g39770S	0.035721037	0.359280662	0.099423767
AT1G50360.1	BjuB01g13790S	0.090907017	0.631931894	0.143855718
AT4G29150.1	BjuB02g71700S	0.060027929	0.474997449	0.126375266
AT2G43680.1	BjuA09g15340S	0.168841992	0.759270061	0.222374094
AT4G10640.1	BjuA03g33400S	0.096690162	0.45066585	0.214549566
AT3G09710.2	BjuA02g41150S	0.255166735	1.081119177	0.236020913
AT2G43680.1	BjuB08g02390S	0.161986284	0.680127946	0.23817031
AT5G13460.1	BjuA02g38690S	0.184165026	0.42993945	0.428351077
AT3G49260.3	BjuB06g48510S	0.061966306	0.437547237	0.141621979
AT1G51960.1	BjuB01g10540S	0.288320823	1.038805579	0.277550322
AT4G14750.3	BjuA08g09760S	0.055524119	0.3750492	0.148044894
AT1G14380.3	BjuB06g23720S	0.176505927	0.309640544	0.570034933
AT1G14380.3	BjuA06g33380S	0.182561539	0.307763029	0.593188661
AT5G03960.3	BjuA10g28160S	0.090375575	0.449115666	0.201230067
AT3G09700.1	BjuA01g03500S	0.07227045	0.401638677	0.17993897
AT2G33990.1	BjuB06g36800S	0.1029444	0.391942849	0.262651558
AT4G23060.1	BjuA08g13600S	0.124306781	0.445074997	0.27929401
AT3G49380.1	BjuB08g10630S	0.120773739	0.433330549	0.278710419
AT1G18840.3	BjuA08g25050S	0.123277091	0.380200926	0.324241953
AT2G26410.1	BjuA03g35120S	0.147485045	0.518001946	0.284719095
AT1G51960.1	BjuA03g24220S	0.278936259	0.775305462	0.35977595
AT3G22190.2	BjuA01g12110S	0.097693949	0.34574389	0.282561606
AT1G18840.3	BjuB03g52360S	0.116513944	0.320524662	0.363510076
AT2G43680.1	BjuB01g48350S	0.147697086	0.351843326	0.419780837
AT4G14750.3	BjuB05g44740S	0.098729246	0.457961858	0.215583993
AT1G74690.1	BjuA02g21850S	0.096762875	0.353307162	0.273877479
AT3G15050.1	BjuA01g07120S	0.091438645	0.353143109	0.258928016

AT2G26140.1	BjuB01g36480S	0.057594515	0.585040873	0.098445284
AT5G03940.1	BjuA02g41590S	0.078870944	0.498992734	0.158060306
AT4G14750.3	BjuB07g17010S	0.063249477	0.405135139	0.156119454
AT2G26140.1	BjuA04g16910S	0.057874943	0.77337294	0.074834456
AT1G19860.2	BjuA06g28870S	0.156357896	0.344680333	0.453631616
AT4G23060.1	BjuA01g32080S	0.093457343	0.451539182	0.206975046
AT3G09710.2	BjuA03g56710S	0.260942781	0.946463327	0.275703002
AT1G17480.1	BjuA06g30710S	0.114533727	0.433377251	0.264281817
AT4G10640.1	BjuB08g33050S	0.086950471	0.467534164	0.185976722
AT2G33990.1	BjuB01g42220S	0.122550427	0.388065518	0.31579829
AT3G22190.2	BjuA03g21940S	0.078235969	0.396758723	0.197187772
AT1G51960.1	BjuB07g44480S	0.273089028	0.849938232	0.321304558
AT1G01110.2	BjuA10g00670S	0.080081958	0.432567365	0.185131761
AT1G14380.3	BjuB04g41980S	0.249001297	0.656178036	0.37947216
AT4G23060.1	BjuB03g41340S	0.121344907	0.457635662	0.265156143
AT2G43680.1	BjuA05g03410S	0.098130365	0.400377113	0.245094841
AT3G51380.1	BjuB02g63340S	0.078270011	0.452468139	0.172984581
AT3G51380.1	BjuA03g16890S	0.080475285	0.510504755	0.15763866
AT3G09710.2	BjuB02g47200S	0.243261092	1.000990202	0.243020453
AT3G51380.1	BjuB08g08840S	0.080444428	0.296674962	0.271153412
AT1G51960.1	BjuA05g16310S	0.198107355	0.581390446	0.340747524
AT3G09710.2	BjuB05g36910S	0.241322683	0.921865063	0.26177658
AT1G19860.2	BjuB03g51860S	0.095306213	0.248485385	0.383548567
AT5G03040.1	BjuB08g60880S	0.075034549	0.484340783	0.154920981
AT1G01110.2	BjuB03g28260S	0.073959131	0.420421614	0.175916576
AT1G19860.2	BjuB04g25360S	0.129701565	0.310285753	0.418006835
AT1G01110.2	BjuB08g29120S	0.156361706	1.125669933	0.138905465
AT3G09710.2	BjuA10g28720S	0.297659323	1.025874224	0.290151868
AT5G13460.1	BjuA03g52650S	0.073713673	0.410831023	0.17942577
AT5G62070.1	BjuA03g54960S	0.252147973	1.29711981	0.194390658
AT3G22190.2	BjuB07g41180S	0.08224039	0.378311181	0.217388209
AT2G02790.1	BjuA09g49730S	0.135170986	0.414747024	0.325911888
AT5G07240.2	BjuA06g17780S	0.17403653	1.157971673	0.150294289
AT1G17480.1	BjuB04g27330S	0.084008288	0.435450523	0.192922693
AT1G51960.1	BjuB07g03270S	0.202462508	0.568474418	0.356150606
AT2G33990.1	BjuA05g09780S	0.104678724	0.475555481	0.220118847
AT3G51380.1	BjuA09g21520S	0.077843348	0.347618764	0.223933101
AT3G22190.2	BjuA05g23040S	0.071482635	0.367490581	0.194515557
AT5G03960.3	BjuB02g46670S	0.109407682	0.421818629	0.259371385
AT3G49260.3	BjuA06g18280S	0.078342661	0.432591978	0.181100587
AT1G14380.3	BjuB04g46420S	0.239466889	0.620910851	0.385670324
AT4G29150.1	BjuA03g08510S	0.069131887	0.512426089	0.134910944
AT1G72670.1	BjuB03g16810S	0.043756049	0.489466616	0.089395369
AT1G51960.1	BjuA05g28520S	0.279976804	1.120912519	0.249775785
AT1G19860.2	BjuA08g24640S	0.091165447	0.28935467	0.315064717
AT3G52290.1	BjuA04g06090S	0.099125383	0.42280571	0.234446651
AT3G15050.1	BjuB07g45330S	0.083935688	0.362089803	0.231809034
AT5G07240.2	BjuA10g25440S	0.097345842	0.559302727	0.174048574
AT5G13460.1	BjuB08g56090S	0.069961585	0.448992253	0.155819136
AT1G18840.3	BjuB04g26240S	0.118239564	0.3510076	0.336857562
AT3G49260.3	BjuB08g10820S	0.066722035	0.431827148	0.154510978
