# Supplementary material for: Structure, evolution, phylogeny, and analysis of domain-deficient genes in the IQD gene family of Brassica juncea
Source: Sci Rep. 2026 Mar 2;16:11773. doi: 10.1038/s41598-026-42340-2 (PMC13065986; doi:10.1038/s41598-026-42340-2)
Supplement: Supplementary file 4 — Supplementary Material 4 [file 41598_2026_42340_MOESM4_ESM.pdf]

Table S3 Renamed *IQD* gene

gene	rename gene
BjuA01g03500S	BjIQD1
BjuA02g21850S	BjIQD2
BjuA03g08510S	BjIQD3
BjuA03g12230S	BjIQD4
BjuA04g06090S	BjIQD5
BjuA05g03410S	BjIQD6
BjuA05g09780S	BjIQD7
BjuA05g23040S	BjIQD8
BjuA05g28520S	BjIQD9
BjuA06g17780S	BjIQD10
BjuA06g33380S	BjIQD11
BjuA08g08190S	BjIQD12
BjuA08g24640S	BjIQD13
BjuA09g21520S	BjIQD14
BjuA10g25440S	BjIQD15
BjuA10g28720S	BjIQD16
BjuB02g46670S	BjIQD17
BjuB03g16810S	BjIQD18
BjuB03g28260S	BjIQD19
BjuB04g26240S	BjIQD20
BjuB04g27330S	BjIQD21
BjuB04g46420S	BjIQD22
BjuB07g03270S	BjIQD23
BjuB07g17010S	BjIQD24
BjuB07g45330S	BjIQD25
BjuB08g02390S	BjIQD26
BjuB08g10630S	BjIQD27
BjuB08g29120S	BjIQD28
BjuB08g33050S	BjIQD29
BjuB08g35820S	BjIQD30
BjuB08g56090S	BjIQD31
