# Supplementary material for: Structure, evolution, phylogeny, and analysis of domain-deficient genes in the IQD gene family of Brassica juncea
Source: Sci Rep. 2026 Mar 2;16:11773. doi: 10.1038/s41598-026-42340-2 (PMC13065986; doi:10.1038/s41598-026-42340-2)
Supplement: Supplementary file 6 — Supplementary Material 6 [file 41598_2026_42340_MOESM6_ESM.pdf]

Table S5 Expression of the *BjIQD* Genes in Different Tissues and Organs

Gene ID	Bud	Root	Stem	Leaf	Seed	Seed coat
BjuB02g44020S	3.089136084	9.054982318	1.918012679	1.969011036	1.397044514	0.134932604
BjuA10g25440S	9.890499223	24.26163241	6.636490895	3.58389539	6.793583113	10.56312739
BjuB08g58260S	0	0	0	0	0	0
BjuA03g54960S	17.87918379	25.8754865	16.25374881	7.92926066	12.93458442	10.28794244
BjuB06g48110S	72.21036131	166.8577451	77.61724758	103.1728966	39.30653963	22.0484372
BjuA06g17780S	11.44252747	19.49575918	9.926984378	15.04274283	2.32277251	1.07801383
BjuA08g13600S	2.690719922	59.24538845	11.37669658	17.44538742	0	0
BjuB03g41340S	3.408655483	33.75018389	1.146157244	0.307528977	0	0.67438014
BjuB05g50960S	17.70927337	20.29964041	10.03333347	31.83541619	2.527158424	75.42047053
BjuA01g32080S	5.407745007	4.56339257	0.440811937	22.60542549	8.332871969	257.619849
BjuB02g68480S	3.692095801	6.333447046	2.070897748	0.659152183	0.937656003	1.626136303
BjuA03g12230S	1.83928582	0.644985485	1.656718198	0.062688599	1.038634341	0.257755871
BjuA08g09760S	21.70756029	0.123943996	4.579546239	0.662562431	1.341686638	0.163454943
BjuB07g17010S	39.48048401	0.398854661	6.309014069	5.496186491	1.177521492	1.948154838
BjuB05g44740S	0	0	0	0	0	0
BjuA01g24750S	4.521298962	0	0	0	0	0
BjuB08g08840S	4.974712655	0	0	0	4.757502939	1.349326036
BjuA09g21520S	10.26934583	0	0	0.156721498	3.029350162	0.515511742
BjuB02g63340S	0.15730734	0	0	0	0.296101896	0
BjuA03g16890S	0	0	0.159299827	0	2.308076314	0.773267613
BjuA08g02200S	0	0	0	0	0	0
BjuB07g03270S	0	0	0	0	0.069671034	4.730578337
BjuA05g16310S	0.43342687	0.138173216	0	0	3.875267104	0.242960217
BjuB02g71700S	7.580087018	1.812355909	1.564034663	0	9.127392696	4.358417454
BjuA03g08510S	4.204081875	0	0.327702501	0.053733085	5.292089263	2.43026964
BjuA01g08250S	1.272813762	2.521529845	0.472448155	0	3.465405454	1.223115312
BjuB07g44480S	10.20065756	0.537009798	2.285687534	0.191378111	13.21159142	3.737712336
BjuA03g24220S	0.831121133	0.611434952	0.066445917	0	2.767840179	0.322539432
BjuB01g10540S	9.651460751	1.667434067	2.070897748	0	7.32543752	0.41885329
BjuA05g28520S	5.249961483	5.319218243	6.104646404	0.212596119	7.191413863	0.437064303
BjuA06g28870S	124.802272	356.7356085	238.5674205	39.23694716	92.64956091	209.0915625
BjuB04g25360S	59.69357322	198.4871068	109.7239466	13.72749419	49.18451718	214.4373355
BjuB03g51860S	69.39939425	166.0229823	111.8621515	38.56113348	67.51123218	123.4629666
BjuA08g24640S	66.12346061	166.905739	98.54405282	34.16760838	71.45633299	89.25608485
BjuA02g09520S	44.9696946	50.07110007	41.4939512	10.61680314	52.50873614	52.32207707
BjuB04g46420S	84.69672335	83.6748732	66.72593912	9.366653691	76.49811698	104.1149607
BjuA06g04950S	53.71570078	35.84373884	22.88582813	10.5185638	46.52518249	62.41888102
BjuB04g41980S	61.85168598	50.64904804	36.4546463	14.34584556	122.2827415	100.857103
BjuA09g49730S	18.37155374	14.03845207	12.28146309	1.699127284	64.64395977	43.23730335
BjuB06g23720S	48.63825702	97.43614725	59.58011408	9.26311662	43.81940225	102.8349867
BjuA06g33380S	51.03701034	109.3705325	73.78108803	11.54983399	65.95924983	149.1233259
BjuB05g04220S	80.32506044	37.87457382	37.01729724	15.02567363	39.85038011	63.66570012
BjuA02g21850S	82.65137382	46.87871778	55.58289555	22.57416459	36.0059905	44.23090745
BjuB04g23570S	5.16589536	6.916763539	3.834995829	1.811003978	4.861920013	3.599035679
BjuA08g25050S	113.170633	134.0084057	159.3187267	19.06158424	79.81200754	113.3033646
BjuB03g52360S	97.65942905	165.6757898	259.7853897	8.345891826	77.18151577	138.9583639
BjuA06g29740S	75.98897895	82.80867369	114.5582981	42.41453635	58.0494307	185.4377749
BjuB04g26240S	129.9148885	134.4957612	148.920558	81.85737981	116.3526915	234.5578426
BjuA06g26760S	0.970545654	0.253147608	0.458501346	0.360864261	1.328634336	0.074188037
BjuB08g10820S	0.6153493	1.681446118	5.847240699	0.359537554	0.082734353	0.443491719

BjuB06g48510S	15.67256993	22.67550194	42.49691008	8.115276229	127.0799665	19.68258487
BjuA06g18280S	30.08122293	9.999481719	33.57971832	9.902102396	181.4011484	203.2834635
BjuA02g41150S	143.3048892	201.1592459	235.2539841	80.19125615	89.11482648	160.1024817
BjuB05g36910S	131.1931343	157.2259987	161.4831361	48.89710741	53.83914617	198.5459431
BjuA03g56710S	13.368611	15.48864361	11.75052524	5.702058851	3.522950348	16.18673929
BjuA10g28720S	102.4599603	100.3258081	143.0770035	25.59295409	23.84439264	48.6796641
BjuB08g60880S	56.22754231	50.6483098	68.727919	70.48059622	26.89900657	51.28334973
BjuB02g47200S	90.61286455	44.13180898	56.82341381	13.86411887	32.10900067	40.14862026
BjuB07g48310S	21.35615203	10.94520824	5.416194109	6.686783919	17.02206281	9.365129977
BjuA01g03500S	21.48323184	15.2064183	7.568763342	3.474921339	16.67790169	8.572672418
BjuA03g27630S	0.503383488	0	0	3.25980716	0	0
BjuB03g39770S	118.8317454	221.8595185	83.46108659	56.37006156	151.0723045	96.80726914
BjuB02g20000S	5.661104021	0.071238839	0.696750644	0	1.822733164	0.250529071
BjuA03g35120S	15.65377788	0.493546774	0	0.263833313	0	0
BjuB08g35820S	16.8288795	0.873923588	0.158285178	3.581635256	12.68523152	0.448199699
BjuB06g02440S	18.48858312	6.290733731	3.140989892	8.36158714	13.88706883	0.597916965
BjuA04g06090S	27.80920192	3.894131735	3.96171743	16.2990358	15.57324317	6.555964543
BjuB07g14640S	20.28595292	12.10283451	15.39954814	3.381182959	3.471322222	7.05812353
BjuA08g08190S	51.26312525	16.19793094	21.36789949	14.90991343	1.278621822	1.599258017
BjuA07g32910S	9.693533378	7.725563247	9.906727063	1.156350513	7.906720886	13.31274242
BjuB03g16810S	12.87500074	5.141842331	3.38205786	0.289331996	12.2505589	12.49124605
BjuA06g30710S	4.059261032	5.903048978	3.06300225	0.625428118	1.099020059	0.84160289
BjuB04g27330S	3.080024391	7.364164017	4.717434869	0.497258719	1.907096954	0.681523998
BjuB01g36480S	154.2045171	99.56807232	66.64895833	79.27744135	118.4213417	244.2258005
BjuA04g16910S	234.6132988	140.530735	161.5300243	122.5028595	142.5237124	350.9099395
BjuB01g16160S	99.83772506	114.8732522	76.44155897	61.69328025	77.97349916	127.6841188
BjuA05g23040S	34.92470674	55.90874543	26.41617599	15.20815545	27.19940719	50.42070159
BjuB07g41180S	3.897671519	9.462482984	3.973007591	2.682443196	1.90470561	8.508367777
BjuA03g21940S	9.69962994	24.48566253	9.143208924	4.612934661	3.821390501	7.586776578
BjuA01g12110S	5.429923478	20.95443609	3.663261354	1.753282736	0.806907158	4.725866606
BjuA01g07120S	3.248248166	103.4654075	204.6203269	0.27677608	0	3.717520523
BjuB07g45330S	9.936920796	162.2515435	329.7299531	0.423351579	0	0
BjuB01g42220S	10.38546236	21.36625472	18.57532525	4.321714038	9.888008754	16.8556718
BjuB06g36800S	41.46678167	174.5977294	146.6419486	27.97266955	28.38310062	32.24038299
BjuA05g09780S	32.56804375	88.61221044	75.40924212	17.28259831	47.91745503	61.87474122
BjuA03g31310S	28.37505815	5.789282838	17.85166286	0.696319567	22.57495844	25.70380698
BjuB08g29120S	26.77770795	24.73217725	30.57181423	2.286555382	7.123487328	28.06015711
BjuA10g00670S	33.94265631	1.298479526	9.877594304	2.930741453	21.43889813	6.278835443
BjuB03g28260S	43.76112112	2.516881611	18.13012368	3.382818751	20.59304881	6.006198125
BjuA06g26870S	3.059283486	0	0.334690545	0	6.137384744	1.692336526
BjuB08g10630S	5.511622594	0.077517516	0.168479817	0	6.941832914	1.771962394
BjuA03g33400S	0.872653856	0	0	0	1.642609056	0.953884865
BjuB08g33050S	6.808474969	0.232552548	0.084239908	0	1.67824532	1.363047995
BjuA04g27580S	15.49768608	79.25038108	39.3799286	7.890803047	16.25270404	16.96688249
BjuB01g48350S	35.14003962	116.7454589	48.19238969	7.720595906	33.8922785	18.15143975
BjuA05g03410S	103.7817024	201.4553529	225.8075044	53.69278525	68.42003723	23.62332557
BjuA09g15340S	18.27570624	202.6774454	437.4210899	6.61823906	1.433359494	3.841711707
BjuB08g02390S	16.74285012	152.04185	286.7011996	3.883327322	5.739209895	6.296854834
BjuB01g13790S	114.0132906	87.98474396	116.0712933	174.41956	56.25936015	99.4214688
BjuB05g41800S	8.291619142	2.858458401	2.505118243	0.098582878	1.179631745	0.081068379
BjuA02g38690S	17.93303675	5.797436758	12.0753756	4.734755119	11.96501885	8.141092121
BjuA03g52650S	26.93940632	9.833096901	21.53733658	4.15625413	9.001497624	1.206297476
BjuB08g56090S	55.97281167	31.18318256	34.82873485	9.909072902	30.00499208	24.52195627
BjuB05g37620S	0.86948057	0	0.150610745	0.148173053	0	0
BjuA02g41590S	250.8867004	116.524216	241.7468577	470.9941963	135.8498079	239.0420366
BjuA10g28160S	4.128468892	0.082257796	0.268173809	0.087944438	0	0

BjuB02g46670S	3.702304361	0	0.64131027	0.157732605	0	0
