# Supplementary material for: Structure, evolution, phylogeny, and analysis of domain-deficient genes in the IQD gene family of Brassica juncea
Source: Sci Rep. 2026 Mar 2;16:11773. doi: 10.1038/s41598-026-42340-2 (PMC13065986; doi:10.1038/s41598-026-42340-2)
Supplement: Supplementary file 7 — Supplementary Material 7 [file 41598_2026_42340_MOESM7_ESM.pdf]

Table S6. Specific primers of *BjIQD* genes for qRT-PCR

**Gene name**	**Sequence(5’-3’)**
BjuA06g28870S	CCCAACCCAATCTGGCGT
	ATGCTGCCGCCTTGTTCT
BjuA09g15340S	GCCAGTAGCCTTGCGTCT
	TGCATAGGCCATGGACCG
BjuA05g03410S	CGAGCCGCTAGAGCAGAC
	CGGAGGAGGAGGAGCACT
BjuB07g45330S	ATCCAAGGCCACACGGTG
	ACGCCGTGCTCTGATCTG
BjuA02g41590S	AGGCAGCTGCTGAGGTTG
	GTGCTCGCAACGCTTTCC
BjuA04g16910S	CAGTGGCAGCGAGTTCGA
	GGTCCTTGGGGTTACGGC
Actin7	TGTGCTTGACTCTGGTGATGGT
	GACGGAGGATAGCGTGAGGAAG
